# Recent Trends on Electrochemical Sensors Based on Ordered Mesoporous Carbon

**DOI:** 10.3390/s17081863

**Published:** 2017-08-11

**Authors:** Alain Walcarius

**Affiliations:** Laboratoire de Chimie Physique et Microbiologie Pour l’Environnement (LCPME), UMR 7564, CNRS—Université de Lorraine, 405 rue de Vandoeuvre, 54600 Villers-les-Nancy, France; alain.walcarius@univ-lorraine.fr; Tel.: +33-3-7274-7375

**Keywords:** ordered mesoporous carbon, electrochemical sensors, electrochemical biosensors, modified electrodes, electrocatalysis, functionalization, carbon composite, carbon nanomaterials, template, preconcentration

## Abstract

The past decade has seen an increasing number of extensive studies devoted to the exploitation of ordered mesoporous carbon (OMC) materials in electrochemistry, notably in the fields of energy and sensing. The present review summarizes the recent achievements made in field of electroanalysis using electrodes modified with such nanomaterials. On the basis of comprehensive tables, the interest in OMC for designing electrochemical sensors is illustrated through the various applications developed to date. They include voltammetric detection after preconcentration, electrocatalysis (intrinsically due to OMC or based on suitable catalysts deposited onto OMC), electrochemical biosensors, as well as electrochemiluminescence and potentiometric sensors.

## 1. Introduction

Carbon is a traditional electrode material that has been commonly used for over a century. Various kinds of carbon-based electrodes have been exploited for electrochemical purposes (electrosynthesis [1], electroanalysis [2]) and the most popular ones in the field of sensors and biosensors are certainly glassy carbon electrodes [3] and carbon paste electrodes [4], among some others [5]. The main reasons for this success are related to the attractive properties of some carbon forms, mainly graphite-containing ones, including a wide potential window and relatively inert electrochemistry, electrocatalytic activity for a variety of redox reactions, and low cost [5].

More recently, the emergence of nanoscale and multi-dimensional forms of carbon such as 0D fullerenes, 1D carbon nanotubes, 2D graphene and related materials, or 3D nanostructured porous carbon materials, has contributed to expand considerably the development of carbon electrodes. Thanks to their additional properties originating from their nanostructures, i.e., ultra-high conducting surface area, ordered structure at the nanoscale, nanoelectrocatalysis promoting electron-transfer reactions (most likely due to the presence of edge-plane-like sites [6]), these advanced materials have been the subject of numerous investigations in the past few decades [7,8,9,10,11,12,13,14,15]. They are especially promising in the field of energy conversion and storage [7,8,9], photovoltaics [10,11] or electrochemical sensors [12,13,14] and biosensors [15]. 

Ordered mesoporous carbons (OMCs) constitute a subclass of 3D nanostructured porous carbon materials. Since their discovery in 1999 [16], the early stages of their development primarily focused on their synthesis by the so-called hard template route (i.e., using mesoporous silica as a scaffold) [17,18,19]. Then, soft template syntheses were developed via self-assembly of copolymer molecular arrays and carbon precursors, and subsequent carbonization [18,19,20]. These materials offer attractive features likely to be exploited in electrochemistry, such as good electronic conductivity, chemical inertness, great porosity (high specific surface area, large pore volume and size) and widely open ordered structure made of uniform and tunable pore sizes (ensuring fast mass transport rates) [21]. Note that the synthetic route using soft-carbon sources generates carbon materials that can be converted into highly ordered graphite at high temperature, leading to OMCs with graphitic pore walls [20], which could be even more interesting for electrochemical purposes, and their physical or chemical properties can be further improved via the incorporation of various components in/on the mesopore walls, which has contributed to extend the scope of their applications [19]. The field of electrochemical sensors based on template-based ordered porous materials is rather young (pioneering works dating from 2007 [22,23,24,25,26]) but it underwent rapid development in recent years, in parallel to the emergence of other types of mesoporous materials in electroanalysis [27,28,29].

The present review aims at describing the development of electrochemical sensors and biosensors utilizing OMC materials for their elaboration, with special emphasis to the progress made in the past five years, as the corresponding state-of-the-art up to 2012 has been described in two reviews [30,31], and a book chapter on the topic is available [32]. Nevertheless, a comprehensive overview of all applications is provided through extensive tables, to enable the reader following the entire evolution of the field, whereas only the most recent advances are discussed in the manuscript.

## 2. Ordered Mesoporous Carbon Materials

### 2.1. Synthesis and Characterization

**Figure 1:**
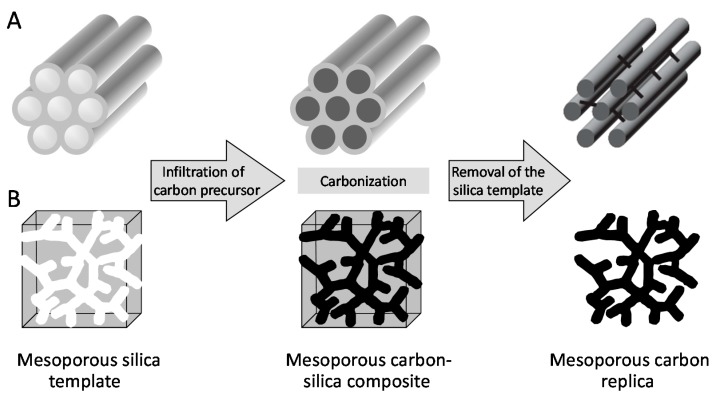
Illustration of the hard template casting route to OMC: (**A**) Hexagonal CMK-3; (**B**) Cubic CMK-1.

The conventional strategy applied to generate OMC materials involves the use of mesoporous silica (with hexagonal or cubic structure) as hard templates [16,33]. The general procedure is illustrated in Figure 1, showing that the pores of the mesoporous silica template are first impregnated with a carbon source (most often sucrose), followed by polymerization and carbonization upon heat treatment (pyrolysis) to give the corresponding mesoporous carbon-silica composite, and final dissolution of the silica framework leads to the free mesoporous carbon replica. Cubic and hexagonal mesoporous carbons obtained by nanocasting from MCM-48 and SBA-15 materials were respectively designed as CMK-1 and CMK-3. The 3D interconnected mesostructure of cubic mesoporous silica ensures an intrinsic 3D pore structure of the mesoporous carbon replica CMK-1, while the mechanical stability of the hexagonal CMK-3 is due to the existence of complementary micropores connecting the hexagonally packed mesopores in SBA-15 silica [33]. Other ordered mesoporous silicas were also used as hard templates for OMC [34].

**Figure 2:**
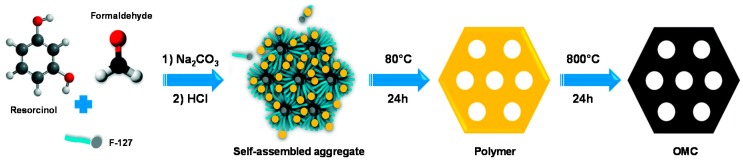
Schematic description of the soft template route to OMC using resorcinol-formaldehyde as carbon source and F127 as copolymer template (adapted from [37]).

Later on, efforts have been made to develop cost-effective strategies for mesoporous carbons in order to circumvent the main disadvantage of the hard template route (due to several steps starting with the synthesis of a mesoporous silica/surfactant mesophase, followed by surfactant removal, then introduction of the carbon precursor into the mesoporous silica and its carbonization, and finally the etching of the silica template with HF or NaOH). A more direct method is based on a soft templating approach [35,36], involving the self-assembly of supramolecular aggregates of block copolymers (acting as template, e.g., F127, P123) and carbon precursors (thermosetting agent, e.g., resorcinol-formaldehyde mixture or phenolic resin), followed by thermopolymerization of the precursors to give a highly cross-linked composite, template removal and carbonization (see illustration in Figure 2 [37]). The driving force of the self-assembly process is thought to be hydrogen bonding or van der Waals forces between the aggregated template and carbon precursors, these latter much better carbon yields in carbonization reactions than direct pyrolysis of block copolymers.

More recent developments concern the preparation of OMC with graphitic pore walls (instead of the amorphous carbon usually obtained from carbonization reactions), the control of pore size and OMC morphology (particles, monoliths, films), as well as functionalization and modification (via either post-synthesis surface treatment or heteroatom doping). More information can be found in selected reviews [18,19,20,34,36]. From the electrochemical point of view, at least two of these advances are of particular interest: the graphitic mesoporous carbons with high electrical conductivity and the continuous thin film morphology (likely to circumvent the poor mechanical stability of OMC particle layers on electrodes). Functionalized OMC might be also attractive for expanding the scope of applications. To date, however, most OMC materials used for electroanalytical purposes are CMK-3 and CMK-1 materials, modified or not, and only few recent investigations are dealing with the new generations of OMC (see below).

### 2.2. Properties and Electrochemical Characteristics

#### 2.2.1. Physico-Chemical Characterization and Properties of OMC

**Figure 3:**
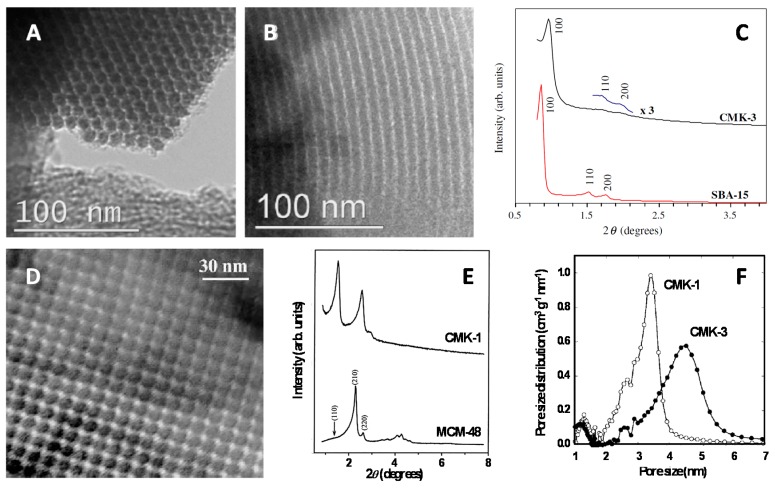
(**A**,**B**,**D**) TEM micrographs and (**C**,**E**) XRD diffractograms of (**A**–**C**) hexagonal CMK-3 and (**D**,**E**) cubic CMK-1 materials. TEM views of CMK-3 show both the hexagonal packing (**A**) and the long mesopores (**B**), while XRD data include also the diffractograms corresponding to the mother SBA-15 (**C**) and MCM-48 (**E**) mesoporous silica templates. (**F**) Pore size distributions for CMK-3 and CMK-1. Data have been reproduced/adapted from [38] (**A**,**B**), [40] (**C**), [39] (**D**), [16] (**E**), and [33,41] (**F**).

The main physico-chemical techniques used to characterize OMC are electron microscopies (scanning electron microscopy, SEM, for morphological analysis, and transmission electron microscopy, TEM, for mesostructure examination), X-ray diffraction (XRD, for structure type determination), and N_2_ adsorption-desorption experiments (for textural characterization, pore size distribution and pore volume evaluation from BET analysis). Some typical TEM [38,39] and XRD [16,40] data, as well as pore size distributions [33,41], for CMK-3 and CMK-1 materials are illustrated in Figure 3. Both TEM (A,B,D) and XRD (C,E) confirm the high level of ordering with respectively hexagonal (see TEM views A&B showing respectively the hexagonal packing & parallel mesopores, and XRD data C comparing the diffractograms of the parent SBA-15 template to that of CMK-3 material) and cubic (TEM view D, and XRD data E) mesostructures. They also exhibit great porosity (e.g., specific surface areas in the 900–1500 m^2^∙g^−1^ range and pore volumes extending from 1.1 cm^3^∙g^−1^ to 1.7 cm^3^∙g^−1^, for CMK-3) with quite narrow pore size distributions (see typical data for CMK-1 & CMK-3 on Figure 3F), which are however slightly wider than the mother silica template. OMC materials are thus thought to be effective supports for immobilization of large quantities of reagents.

#### 2.2.2. Electrochemical Characteristics of OMC

**Figure 4:**
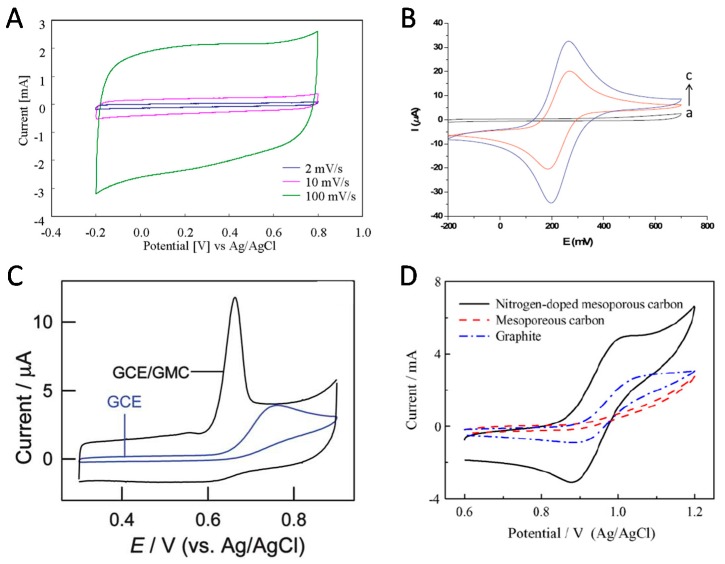
(**A**) Cyclic voltammograms of an OMC (COU-3 type) film electrode at various scan rates (reproduced from [42]). (**B**) Cyclic voltammograms of 1 mM [Fe(CN)_6_]^3−^/[Fe(CN)_6_]^4−^ in 0.1 M KCl recorded at 75 mV∙s^−1^ using screen-printed carbon electrodes (SPCE): (a–c) blank, bare SPCE, and CMK-3/chitosan modified SPCE (reproduced from [43]). (**C**) Cyclic voltammograms of xanthine recorded at bare glassy carbon electrode (GCE) and graphitized mesoporous carbon (GMC) modified GCE (reproduced from [44]). (**D**) Cyclic voltammograms on different carbon electrodes recorded in 3 M H_2_SO_4_ and 1 M VOSO_4_ at 50 mV∙s^−1^ (reproduced from [48]).

Based on the above characteristics of widely open, and thus accessible, mesoporous structures with very large surface areas, it is not so surprising that electrodes modified with OMC were characterized by important capacitive currents (Figure 4A) originating from electroactive areas much larger than flat electrodes of the same geometric surface, being therefore attractive for applications in supercapacitors [42], for instance. However, when applied to the electrochemical transformation of a reversible redox probe in solution (i.e., [Fe(CN)_6_]^3−/4−^ system [43]), the benefit of large electroactive surface area is not overwhelming (compare curves b and c in Figure 4B) because the overall electrochemical processes are diffusion-controlled and the slightly increased currents are simply due to the roughness of the OMC modified electrode surface. Much more improvement can be observed when considering irreversible redox species for which the electrochemical response becomes dominated by the rate of electron transfer; in that case, the presence of OMC is likely to accelerate the kinetics of charge transfer reactions, leading to both higher peak currents and lower overpotentials in comparison to the bare glassy carbon electrode (see Figure 4C for the example of xanthine) [44]. Note that the attractive electronic properties ensuring fast charge transfer processes are strongly dependent on the OMC type (graphitized or not), pore characteristics (more or less open mesostructures), or pre-treatments (e.g., activation to generate oxygen-active sites on the carbon surface) [45,46,47]. In this respect, the emergence of nitrogen-doped OMC has also led to significant improvements in the voltammetric response of irreversible redox systems [48] (example on Figure 4D), which is attributed to increased edge-plane defect sites on the N-doped carbon skeleton [49].

In addition, the widely open 3D structure of OMC promotes fast transport of reagents to the numerous active sites, which contribute to high sensitivities of electrochemical detections, as also reported for other types of mesoporous electrodes [21,28]. Permeability/permselective properties of OMC modified electrodes can be tuned by functionalization of the mesopore walls [50].

#### 2.2.3. OMC Modified Electrodes

Several strategies have been developed to prepare OMC modified electrodes, most of them being based of film technologies [31]:

- Thick layers of OMC particles can be deposited on solid electrode surfaces (mainly glassy carbon but also pyrolytic graphite or screen-printed carbon electrodes) from OMC dispersion in a suitable solvent, with or without an additional polymeric binder (mainly Nafion, but also chitosan), leading to particulate or composite OMC films. Composite OMC-polymer films are usually more mechanically stable than layers made of only OMC particles but the polymeric binder is likely to affect the OMC electrode response via interactions with species in solution. An alternative configuration is the bilayer OMC particles + organic polymer overcoating. These approaches are, by far, the most widely used in designing OMC-based electrochemical sensors.
- Continuous mesoporous thin films can be generated onto current collector substrates, either directly [51], or by transferring a free-standing OMC membrane [52], but their application in the sensors field remains seldom to date.
- A last approach is the dispersion of as-synthesized OMC particles in carbon paste electrodes or the one-step preparation of OMC paste electrodes by mixing OMC particles with mineral oil.

Some post-treatments can be also applied to OMC modified electrodes, such as the electrodeposition of metals or the electropolymerization of conducting polymers or redox-active macromolecules onto the mesopore walls, in order to bring additional properties (electrocatalysis, for instance). Functionalization by self-assembly or adsorption of molecular mediators can be also exploited to achieve this goal.

## 3. Electrochemical Sensors and Biosensors Applications

### 3.1. Electrochemical Sensors Based on Unmodified OMC

OMC materials by themselves are attractive electrode substrates for electrochemical sensing owing to their attractive properties (large conducting surface area, regular and widely open nanostructure, catalytic edge-plane-like sites, or oxygenated surface functions). Table 1 gathers all applications of unmodified OMC-based electrochemical sensors reported to date [23,25,43,44,45,53,54,55,56,57,58,59,60,61,62,63,64,65,66,67,68,69,70,71,72,73,74,75,76,77,78,79,80,81,82,83,84,85,86,87,88,89,90,91,92,93,94,95,96,97,98,99,100,101,102]. They can be classified in two categories: (1) preconcentration electroanalysis and (2) direct detection, mostly via electrocatalysis.

#### 3.1.1. Preconcentration Electroanalysis Using Unmodified OMC

The open-circuit accumulation of analytes via sorption processes (e.g., adsorption, surface complexation) prior to their voltammetric detection is a classical method in electrochemical sensing. The large and easily accessible surface area of OMC offers good opportunities for preconcentration electroanalysis. Several drugs and biologically-relevant molecules, as well as some pesticides, have been detected after accumulation at OMC-modified electrodes. The preconcentration mechanism is not always described but it is possibly due to hydrophobic and/or π–π interactions as most of the investigated species possess aromatic moieties in their molecular structure (i.e., phenol [57,59,72,79,98], nitrophenyl or nitroaromatic groups [64,78,85,87,89,90], catechol [65,97], or other aromatic groups [60,96,99]). Due to the non-specific nature of these interactions, the sensor selectivity is expected to be rather poor (only controlled by the detection potential) and thus limited in real environmental monitoring (except for spiked samples) but useful to the analysis of drug formulations (pharmaceutical tablets) or foodstuffs, for instance. Some examples of metal ions detection after accumulation under cathodic potential and detection by anodic stripping are also available [67,91,92].

These applications were mostly based on OMC film electrodes (Table 1), but also on carbon paste [59,89] and ionic liquid-based carbon paste electrodes [57,60,65,92,97]. In this last case, the ionic liquid serves as a binder but it could also contribute to accumulate the target analyte.

#### 3.1.2. Direct Detection and Electrocatalysis with Unmodified OMC

**Figure 5:**
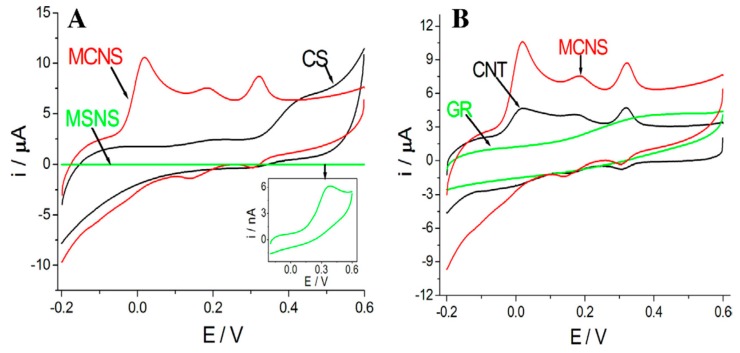
(**A**) Cyclic voltammograms of glassy carbon electrodes (GCE) modified with mesoporous carbon nanospheres (MCNS), non-porous carbon spheres (CS), and mesoporous silica nanospheres (MSNS) in 0.1 M pH 7.4 PBS containing 0.4 mM ascorbic acid (AA), 4 µM dopamine (DA) and 30 µM uric acid (UA). (**B**) Cyclic voltammograms of GCE modified with MCNS, graphene (GR), and carbon nanotubes (CNT) in 0.1 M pH 7.4 PBS containing 0.4 mM AA, 4 µM DA and 30 µM UA (reproduced from [104]).

An attractive feature of OMC is its ability to show faster electron transfer rates in comparison to glassy carbon electrodes, an advantage that can be attributed to the existence of a large amount of edge-plane defect sites and surface oxygen-containing groups [30,31]. This was already mentioned in the pioneering 2007 work on the selective detection of dopamine in the presence of ascorbic acid [23], and then largely exploited in numerous other examples (Table 1). Sometimes, efforts have been directed to the synthesis of highly defective mesoporous carbon to enhance its electrocatalytic properties, giving rise to improvement in heterogeneous electron transfer rates for various redox probes when compared to electrodes based on graphite, multi-walled carbon nanotubes or graphene [103]. This is of particular interest for the one-step analysis of several compounds in mixture for which the OMC resolved clearly the mixed voltammetric signals into well-defined voltammetric peaks [104]. An illustration is given in Figure 5 for the concomitant detection of ascorbic acid (AA), dopamine (DA) and uric acid (UA), using glassy carbon electrodes (GCE) modified with various nano-objects, showing clearly a much better resolution when using mesoporous carbon spheres, as compared to non-porous carbon spheres, and the situation was even worse when operating with GCE covered by mesoporous silica nanospheres for which overlapping response of AA, DA and UA was observed (Figure 5A). The OMC-based sensor performance was better than for GCE modified with either carbon nanotubes or graphene (Figure 5B). In general, the electrocatalytic effect resulted in significant increase in peak currents and decrease in overpotentials. Another nice example is the electrochemical sensing of tea polyphenols using an OMC-modified GCE that succeeded in the simultaneous detection of 1,4-, 1,2- and 1,3-dihydroxy benzene isomers, thanks to the synergetic sorption ability and catalytic properties of the graphitized mesoporous carbon modifier [93]. 

### 3.2. Electrochemical Sensors Based on Functionalized OMC

**Figure 6:**
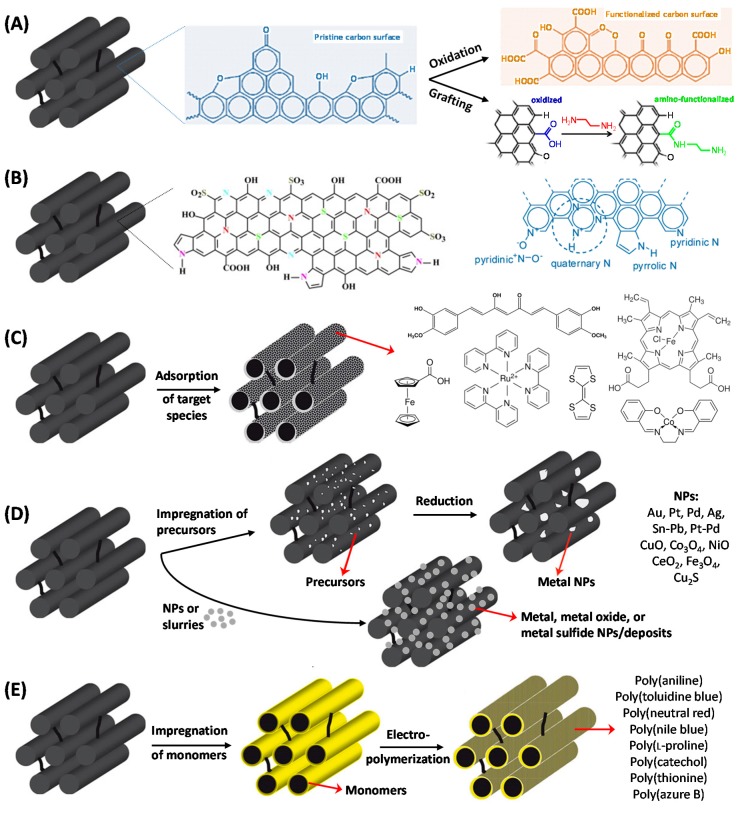
Illustration of the various ways to functionalizing OMC used in electrochemical sensors: (**A**) surface oxidation and grafting; (**B**) bulk N,S-doping; (**C**) adsorption of mediators (**D**) binding of metal nanoparticles or deposition of metal, metal oxide or metal sulfide; (**E**) electropolymerization.

In order to further improve the OMC properties, several modification strategies have been developed. Figure 6 illustrates all the cases used to date in connection to the elaboration of OMC-based electrochemical sensing devices. Various approaches can be distinguished:

- The simplest way is surface oxidation to generate a high density of oxygen-containing groups (carboxylic acid, phenol, carbonyl, etc.) [105,106] or surface grafting of reactive functions (e.g., amine) [50] (Figure 6A). The success of such reactions is easily monitored by surface analysis (using X-ray Photoelectron Spectroscopy, XPS, for example).
- Bulk functionalization by doping is possible by adding a nitrogen- and/or sulfur-containing dopant in the precursor synthesis medium, but this strategy (Figure 6B) has been applied for electroanalytical purposes only very recently (N-doping [107,108,109,110] or dual N,S-doping [111]). XPS and Raman spectroscopy are usually used to evidence these additional sites in OMC.
- Series of redox mediators have been adsorbed onto OMC surfaces (Figure 6C), either via π–π stacking, or hydrophobic interactions, or electrostatic attractions, or combinations of these effects. Examples are available for ferrocene-carboxylic acid [112,113], Ru(bpy)_3_^2+^ [114], metal porphyrins [115,116] and other metal-ligand complexes [117,118], polyoxometalate and related derivatives [24,119], curcumin [120], tetrathiafulvalene [121], metal hexacyanoferrates [122,123,124,125,126]. Non-redox reagents were also immobilized on OMC, such as organic ligands or polymers [127,128,129], as well as ionic liquids [130,131,132] or surfactants [126,133,134]. Due to the electronic conductivity of OMC, the redox-active species can be involved in mediated electrocatalytic schemes in their immobilized form (contrary to non-conducting mesoporous hosts, such as silica-based nanomaterials, for which a certain physical mobility of mediators is necessary to get high sensitivity [135,136], except in case of charge transfer by electron hopping [137,138]).
- Probably the most widely-used approach is the immobilization of noble metal catalysts in the form of nanoparticles (NPs) or electrogenerated deposits [128,132,134,139,140,141,142,143,144,145,146,147,148,149,150,151,152,153,154,155] (or even bimetallic NPs [156,157]), which can be formed by either impregnation of metal precursors and subsequent reduction or from nanoparticles suspensions or slurries, or other NPs/deposits (metal oxides or hydroxides, metal sulfides, etc. [106,158,159,160,161,162,163,164,165,166,167,168,169,170,171,172]) accommodated to OMC by impregnation (Figure 6D).
- The last category is that of conducting and/or redox polymers that have been generated onto OMC by electropolymerization of previously impregnated monomers (Figure 6E), exploiting both the conductivity and large surface area of OMC materials. It was the case of polyaniline [173,174] and a series of polymers derived from phenothiazines [175,176,177], phenoxazine [178] or phenazine [179], as well as poly(catechol) [180] and poly(l-proline) [181].

Finally, more occasional modifiers are cerium 12-tungtophosphoric acid [182], mercaptopropyl-triethoxysilane [183], fullerene [184], carbon nanotubes [185], or Prussian Blue [186]. Ionophores have been also used in connection to macro- and mesoporous carbons for designing membrane-based potentiometric sensors [26,187,188,189].

The electrochemical sensors based on functionalized OMC [24,26,49,50,106,107,108,109,110,111,112,113,114,115,116,117,118,119,120,121,122,123,124,125,126,127,128,129,130,131,132,133,134,139,140,141,142,143,144,145,146,147,148,149,150,151,152,153,154,155,156,157,158,159,160,161,162,163,164,165,166,167,168,169,170,171,172,173,174,175,176,177,178,179,180,181,182,183,184,185,186,187,188,189,190] are reported in Table 2. They are mainly based on either mediated or supported electrocatalysis, along with some other detection schemes, as described below.

#### 3.2.1. Mediated Electrocatalysis Using OMC Modified Electrodes

Mediated electrocatalysis involves the use of a charge transfer cofactor that is likely to lower the overpotential observed for the electrochemical detection of target species exhibiting slow heterogeneous electron transfer rates, which is usually associated to an increase in the current response due to redox recycling of the mediator. OMC materials have been exploited for the immobilization of large quantities of various mediators on electrode surfaces, either in the form of adsorbed molecular or organometallic compounds (Figure 6C) or electropolymerized mediator layers (Figure 6E). The concept has been established in the early stages of OMC modified electrodes development [31] and most recent works concern the extension to other electrocatalysts (such as porphyrin derivatives [115,117], for instance) and to improve the long-term immobilization stability, i.e., by developing binding strategies based on durable chemical grafting instead of the simple mediator adsorption via weak interactions [117]. The large surface areas of OMC indeed enables hosting large amounts of mediators by providing a favorable microenvironment around them to retain their electrocatalytic activity, contributing meanwhile to significant decrease in porosity values (>50%) and pore size (e.g., by 10% for adsorbed species) [115]. Molecular redox mediators immobilized onto OMC have been especially applied to the electrocatalytic detection of hydrogen peroxide and other biologically-relevant molecules (Table 2). The amount of deposited mediator in the form of polymers (based on phenothiazines [175,176,177], phenoxazine [178], phenazine [179], catechol [180] or poly(l-proline) [181]) can be basically controlled by the electropolymerization conditions (time, number of voltammetric scans). The use of an additive, such as an ionic liquid adsorbed onto OMC prior to electropolymerization, was sometimes suggested to enhance the sensor performance [177]. These polymeric mediator-OMC nanocomposites were especially applied to NADH determination [175,176,177,178,179,180].

Besides the strategies of OMC surface modification with mediators, another recent approach is the bulk functionalization via N-doping [49,107,108,109,110] (or even N,S dual-doping [111]) to generate OMC materials containing more catalytically active sites. In the field of electrochemical sensors, they are particularly suited to detection of mixtures (e.g., ascorbic acid, dopamine and uric acid [107,108,109] or hydroquinone and catechol [111]) with well-resolved electrochemical signals attributed to superior electrocatalytic behavior due to increased edge-plane defect sites [49].

#### 3.2.2. Supported Electrocatalysis Using OMC Modified Electrodes

A huge amount of work has been devoted to the development of OMC-based electrochemical sensors bearing noble metal or metal oxide nanoparticles acting as supported electrocatalysts. As shown in Table 2, this has been extensively applied to the non-enzymatic sensing of glucose and hydrogen peroxide, as well as hydrazine and some other analytes. A major interest of the OMC support is its electrical conductivity ensuring a direct electronic connection of each immobilized nanoparticle [149], contrary to non-conductive mesoporous supports (e.g., silica) for which direct electrical wiring to the electrode surface is less easier (it needs a high density of nanoparticles located close to each other to enable electron percolation) [28]. If pioneering works mostly used CMK-3 as OMC support, the most recent trends are based on less common mesoporous carbons with larger pores [117,142,162,163] or cubic mesostructures [134,144]. Another approach is the combination of several modifiers in a single nanocomposite (e.g., polymer and nanoparticles [151], polyoxometalate and metal nanoparticles [119] or three-component ionic liquid-MoS_2_-palladium nanoparticles [132]) in order to induce synergistic effects.

**Figure 7:**
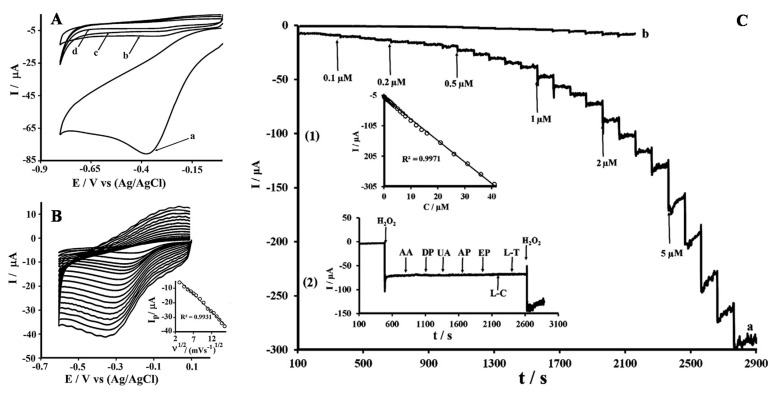
(**A**) Cyclic voltammograms (CV) of (a) glassy carbon electrode (GCE) modified with silver nanoparticles (AgNPs) functionalized OMC (GCE/OMC/AgNPs), (b) GCE/OMC, and (c) bare GCE in 0.1 M phosphate buffer solution (pH 7) containing 0.1 mM H_2_O_2_; (d) CV of GCE/OMC/AgNPs in blank electrolyte. (**B**) CV of 0.025 mM H_2_O_2_ on GCE/OMC/AgNPs recorded at various scan rates (10, 20, 30, 40, 50, 60, 70, 80, 90, 100, 120, 140, 160, 180, 200, 220, 240 mV∙s^−1^) in the same medium as in (**A**) with corresponding plot of peak current versus square root of scan rate in inset. (**C**) Amperometric responses of (a) GCE/OMC/AgNPs and (b) GCE/OMC at −0.2 V to successive addition of H_2_O_2_ (0.1–5 µM); plot of current vs. H_2_O_2_ concentration (inset 1) and current-time curve for GCE/OMC/AgNPs recorded at bare glassy carbon electrode (GCE) and graphitized mesoporous carbon (GMC) modified exposed to 0.01 mM of H_2_O_2_ and 0.1 mM of ascorbic acid (AA), dopamine (DP), uric acid (UA), acetaminophen (AP), epinephrine (EP), l-cysteine (L-C) and l-tyrosine (L-T) and finally 0.01 mM of H_2_O_2_ (inset 2) (reproduced from [148]).

**Figure 8:**
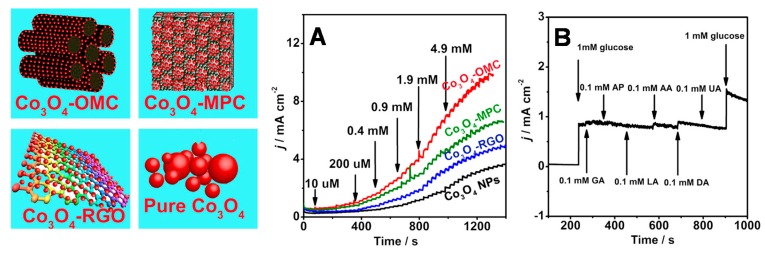
(**A**) Typical *j*–*t* curves obtained at glassy carbon electrodes (GCE) modified with Co_3_O_4_ nanocrystals (GCE/Co_3_O_4_ NPs), or with Co_3_O_4_ nanocrystals-decorated reduced graphene oxide (GCE/Co_3_O_4_-RGO), macroporous carbon (GCE/Co_3_O_4_-MPC), and ordered mesoporous carbon (GCE/Co_3_O_4_-OMC) on the successive injection of glucose in 0.1 M NaOH solution at 0.55 V vs. Ag/AgCl, on which the concentration denoted the final glucose concentration; the corresponding modifiers are illustrated on the left. (**B**) The *j*–*t* curve of GCE/Co_3_O_4_-OMC with the successive addition of 1 mM glucose, 0.1 mM interference substances, and 1 mM glucose at +0.55 V vs. Ag/AgCl (reproduced from [164]).

Rather than describing all the sensor applications of nanoparticles modified OMC materials (which are listed in Table 2), only two illustrative examples are considered hereafter. Figure 7 shows typical voltammetric and amperometric responses to hydrogen peroxide of an OMC modified glassy carbon electrode (GCE) bearing silver nanoparticles (AgNPs). The beneficial effect of AgNPs can be evidenced (Figure 7A) through the dramatic peak current increase in comparison to bare GCE and GCE modified with OMC (without AgNPs). The good electrocatalytic behavior was attributed to the existence of very small and non-aggregated AgNPs uniformly distributed on/in OMC [148]. The intensity of voltammetric current was directly proportional to the square root of potential scan rate (Figure 7B), indicating diffusion-controlled processes owing to fast mass transport through the regular mesoporous structure. From current-time plot recorded upon successive addition of increasing H_2_O_2_ concentrations (Figure 7C), one can see a rapid and sensitive response to variation in the analyte content over a wide range (0.1–41 µM). Also, thanks to the low overpotential (applied potential = −0.2 V), the sensor was extremely selective to H_2_O_2_ in the presence of several common interference species (see bottom left inset in Figure 7C). The amperometric response of GCE/OMC/AgNPs was larger than that of GCE/OMC (i.e., the same electrode but without AgNPs) by several orders of magnitude (compare curves a and b in Figure 7C), confirming the key role played by the nanocatalysts in increasing the amount of active sites for H_2_O_2_ reduction in the AgNPs decorated OMC. The second example concerns the non-enzymatic glucose sensing using ultrafine Co_3_O_4_ nanocrystals embedded mesoporous carbon matrices with specific skeletal structures (Figure 8). In this work [164], the authors have compared the analytical performance of carbon electrodes modified with Co_3_O_4_ nanocrystals alone or immobilized into/onto three kinds of nanocarbons (an ordered mesoporous carbon, OMC, a macroporous carbon, MPC, and reduced graphene oxide, RGO). In all cases, Co_3_O_4_ nanocrystals were electroactive, being reversibly and successively transformed into CoOOH and CoO_2_ [164], and likely to be applied to the direct electrocatalytic oxidation of glucose. However, the sensitivity of the sensor was significantly dependent on the electrode type, being optimal for Co_3_O_4_ on OMC (Figure 8A) as a result of a synergistic effect of three factors: the high number of catalytic sites provided by the uniformly dispersed Co_3_O_4_ nanocrystals in OMC, the fast mass transport processes ensured by the 3D mesostructured, and the improved electron transfer rates in such confined environment (intimate contact between Co_3_O_4_ nanocrystals and the small pore conductive OMC matrix). The glucose sensor was also highly selective with respect to common co-existing interferences (Figure 8B).

#### 3.2.3. Other Electrochemical Sensors Based on Functionalized OMC Modified Electrodes

**Figure 9:**
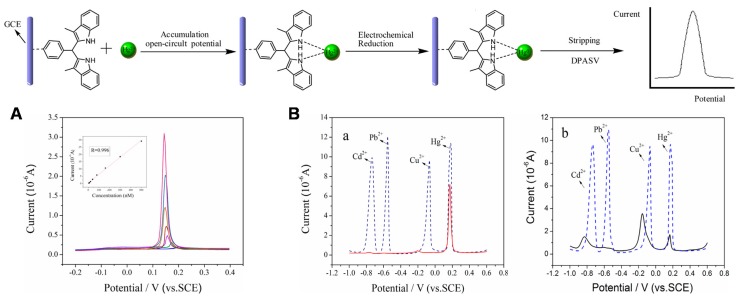
Preconcentration electroanalysis of mercuric ions at a glassy carbon electrode (GCE) modified with bis(indolyl)methane (BIM) on ordered mesoporous carbon nanofibers (OMC) and Nafion (GCE/BIM/OMC/Nafion); accumulation at open-circuit, electrochemical reduction and detection by differential pulse anodic stripping voltammetry (DPASV). (**A**) DPASV curves obtained at GCE/BIM/OMC/Nafion with open-circuit accumulation in 0.1 M phosphate buffer solution (pH 6) containing 5.0–500 nM Hg^2+^; Inset showed the corresponding calibration plot; amplitude of 0.05 V; pulse width of 0.05 s; pulse period of 0.2 s; quiet time of 2 s. (**B**) DPASV curves obtained at (a) GCE/BIM/OMC/Nafion and (b) GCE/OMC/Nafion in the presence of 1.0 µM Hg^2+^ + 10.0 µM Pb^2+^ + 10.0 µM Cu^2+^ + 10.0 µM Cd^2+^; solid lines: 15 min open-circuit accumulation; dash lines: electro-accumulation at −1.2 V for 15 min; other conditions as in part A (reproduced from [127]).

Functionalized OMC were also exploited in some other electrochemical sensing schemes:

- Preconcentration electroanalysis. Ultrasensitive sensors were designed from OMC electrodes modified with selective recognition hosts such as molecularly imprinted polymers for detection of pharmaceuticals (i.e., dimetridazole [128] and ofloxacin [129]) or anchoring ligands for metal ions determination [174]. Figure 9 illustrates an interesting example for mercuric ions detection after open-circuit preconcentration by complexation to bis(indolyl)methane immobilized onto mesoporous carbon nanofibers and subsequent detection by stripping voltammetry in the nanomolar concentration range (Figure 9A). Interestingly, the sensor was highly selective towards mercury recognition in the presence of other metal ions (Cu^2+^, Pb^2+^, Cd^2+^), especially in comparison to the unmodified OMC electrode for which all species were likely to accumulate (see part a in Figure 9B), but only if the preconcentration step was performed at open-circuit for which the organic ligand inhibits the accumulation of Cu^2+^, Pb^2+^ and Cd^2+^, while promoting the enrichment of Hg^2+^ species. In case of accumulation under cathodic potential, whatever the electrode used, the four stripping peaks were observed (see part b in Figure 9B), confirming the critical role played by the organic ligand to ensure high selectivity. Nevertheless, accumulation under potential can be applied as long as differentiation between the various species can be made on the basis of their different stripping peak potentials, and this has been notably exploited for the simultaneous detection of Pb^2+^ and Cd^2+^ in the picomolar concentration range using OMC modified electrode functionalized with bismuth oxide [172]. In that case, both metal ions and bismuth oxide are reduced in the form of an amalgam in the preconcentration step and square wave anodic stripping voltammetry is applied for detection. The interest of OMC for preconcentration electroanalysis applications is similar as for mesoporous silica-based sensors [191], exhibiting faster mass transport rates in comparison to their non-ordered homologs [192].
- Potentiometry. After pioneering works using macroporous carbon as solid contact associated to an ionophore polymer in ion-selective electrodes (ISE) for Ag^+^ or K^+^ sensing [26,187,188], colloid-imprinted mesoporous carbon materials were also exploited for that purpose [189]. An advantage of such hydrophobic intermediate mesoporous layer between the metal electrode and the ionophore-doped ISE membrane is its excellent resistance to the formation of a water layer and no interference caused by light, oxygen and carbon dioxide [189]. Mesoporous carbon was also associated to reference membrane electrode and applied to the potentiometric sensing of chloride ions [130], or as contact layer for pH sensing of a sputtered RuO_2_ thin film [166].
- Electrochemiluminescence (ECL). OMC-based ECL sensors have been also developed recently. A first example concerns electrodeposited polyaniline onto OMC giving rise to a strong ECL emission of luminol originating from the electrochemical reduction of dissolved oxygen [173]. This cathodic ECL response was also applied to H_2_O_2_ sensing. A second example relies on OMC with adsorbed Ru(bpy)_3_^2+^ and tri-*n*-propylamine as coreactant for dopamine detection, offering a successful amplification strategy for ultrasensitive ECL sensing [114].

### 3.3. OMC-Based Electrochemical Biosensors

Carbon nanomaterials have long been recognized as attractive electrode modifiers for building high performance electrochemical biosensors [15,193]. Among them, OMC might be advantageous in some cases, by improving the linear range, detection limit, sensitivity, response time, or lowering overpotentials, with respect to other carbon nanomaterials (such as CNTs, for instance) [194,195,196]. Similar trends were observed for carbon paste-based biosensors [116]. They can serve as hosts for the biomolecules and associated cofactors and mediators, and the abundant interconnected pores in the OMC can facilitate mass transport and offer large accessible surface area for reactants and electrons. The various biosensing applications involving OMC materials [194,195,196,197,198,199,200,201,202,203,204,205,206,207,208,209,210,211,212,213,214,215,216,217,218,219,220,221,222,223,224,225,226,227,228,229,230,231,232,233,234,235,236,237,238,239,240,241,242,243,244,245,246,247,248,249,250,251,252,253,254,255,256,257,258] are summarized in Table 3. They include mainly electrochemical biosensors based on small redox proteins [22,197,198,199,200,201,202,203,204,205,206,207,208], enzymatic biosensors [117,194,195,196,209,210,211,212,213,214,215,216,217,218,219,220,221,222,223,224,225,226,227,228,229,230,231,232,233,234,235,236,237,238,239,240,241,242,243,244,245,246], as well as some immuno- and apta-sensors [247,248,249,250,251,252,253,254,255,256]. They are briefly described hereafter.

#### 3.3.1. Biosensors Based on Small Redox Proteins Immobilized on OMC

The small redox proteins hemoglobin (Hb, ~16 kDa), myoglobin (Mb, ~17 kDa) and cytochrome c (Cyt c, ~12 kDa), have been immobilized onto various kinds of OMC materials by impregnation or adsorption and, after deposition on solid electrode surfaces or dispersion in carbon paste electrodes, they have been successfully applied to the electrocatalytic sensing of hydrogen peroxide (Table 3).

Taking into account the isoelectric point of these proteins (6.8, 7.0 and 10.0, respectively for Hb, Mb and Cyt c [259]), i.e., exhibiting positive surfaces in neutral and acidic media, efforts have been made to functionalize OMC with negatively-charged groups (e.g., carboxylate) in order to enhance the binding strength via favorable electrostatic interactions [22,201,202]. This also contributes to increase the hydrophilic/hydrophobic balance, which can be beneficial to the protein stability (Hb, Mb and Cyt c can be denatured on hydrophobic surfaces [260]). Considering this point, poly(vinyl alcohol) has been used to modify OMC into a highly hydrophilic composite material exhibiting efficient Hb immobilization and good biocompatibility, resulting in improved electron transfer rates and biosensing performance [197]. Similar improvements can be achieved from the modification of OMC with Ni, Pd or polypyrrole nanoparticles embedded in an ionic liquid for Mb adsorption [205]. Actually, in all cases, direct electrochemistry of the proteins is expected to occur on the OMC surface and faster heterogeneous electron transfer kinetics was observed for either graphitized mesoporous carbon and/or OMC materials characterized by pore sizes of the same order of magnitude as the protein dimensions [198,261]. Beneficial effects due to good pore size matching have been also reported for other redox proteins immobilized in OMC [226,262].

#### 3.3.2. Enzymatic OMC-Based Biosensors 

In early 2005, a Korean group reported the immobilization of glucose oxidase (GOD) in mesocellular carbon foam (a multimodal mesoporous carbon) for highly sensitive and fast glucose biosensing [213]. This conductive material exhibited a combination of mesopores containing GOD enzymes and micropores as transport channels, resulting in high enzyme loading and low mass transfer limitations. This was the starting point of huge developments on electrochemical biosensors for glucose based on various types of OMC materials (Table 3). First generation glucose biosensors were especially reported at the beginning [196,214,220,223,226], exploiting the electrocatalytic properties of OMC for the effective detection of the enzymatically-generated H_2_O_2_ product. From a comparative study [263], it was even claimed that OMC shows enhanced electrocatalytic features in comparison to graphene, as explained by different microstructures in these materials, although graphene-based electrochemical sensors and biosensors are now well-established [264,265,266]. OMC materials with hierarchical pore structures and/or large mesopores [214,220,221,222,223,226,231] seem to be the most promising ones (ensuring high enzyme loadings and fast transport of reagents). Graphitized or partially graphitic mesoporous carbons are also attractive because of their high conductivity [200,232]. The bioelectrode configuration most often implied the use of Nafion to confine OMC particles onto the electrode surface, this polymer offering at the same time a way of durable enzyme immobilization. Other strategies for improved performance and lifetime involve ship-in-a-bottle approaches (e.g., enzyme cross-linking in bottleneck pore structures [234,267]) or the covalent bonding of GOD to the OMC surface [230]. Metal nanoparticles-decorated OMC enable to further improve charge transfer kinetics by providing a higher number of active sites (Pt, Au and Pd NPs have been used for that purpose [142,216,217,218,219,224,232], and even alloyed NiFe_2_ NPs [233]), which could also lead to electrical contacting of redox proteins for advanced bioelectrocatalysis [268,269]. Note that if the overall conductivity of the composite continuously increases with NPs loading amounts (e.g., from 5% to 50% Pt NPs, for instance), due to better interconnectivity, the dependence of the electrocatalytic response (i.e., to H_2_O_2_) on NPs loading follows an inverted V-shaped profile, with an optimal situation where Pt NPs are well-dispersed on OMC with little interconnection [235]. Other NPs such as iron oxides were also used in OMC-based glucose biosensors [215,225] but their catalytic role is less explicit. Recently, second generation glucose biosensors integrating OMC functionalized with suitable mediators have appeared [117,234], but this remains underexplored most probably because the intrinsic electrocatalytic properties of OMC and metal PPS-OMC materials are satisfactory by themselves.

**Figure 10:**
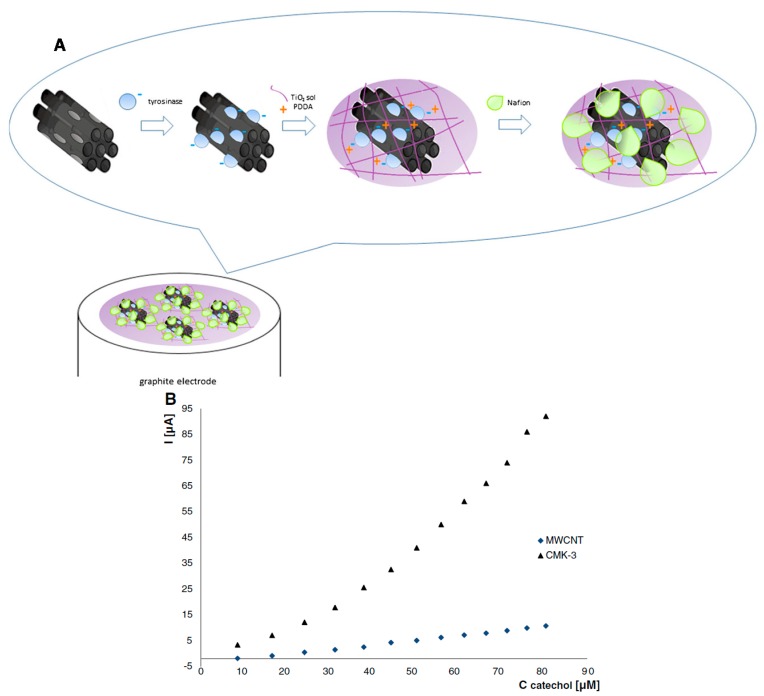
(**A**) Schematic illustration of a tyrosinase (TYR) OMC-based biosensor (TYR/TiO_2_/CMK-3/PDDA/Nafion, with PDDA = poly(diallyl-dimethyl-ammonium chloride)). (**B**) Corresponding calibration curve for catechol and comparison to a similar system based on carbon nanotubes (reproduced from [246]).

**Figure 11:**
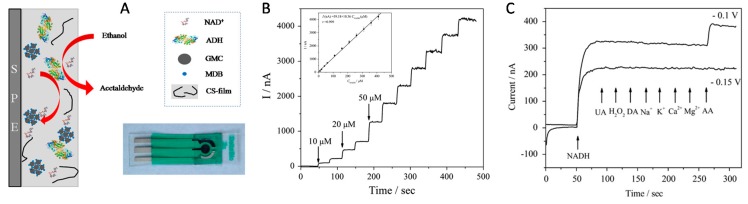
(**A**) Schematic illustration of an alcohol dehydrogenase (ADH)-based biosensor constructed from ADH/NAD^+^/Meldola’s blue (MDB)/graphitized mesoporous carbon (GMC)/chitosan (CS-film) nanobiocomposite on a screen-printed electrode (SPE, shown on the photograph). (**B**) Typical amperometric response upon successive addition of NADH (10, 20 and 50 µM) in a stirred Tris-HCl buffer solution (0.1 M, pH 8.0, 300 rpm) at a working potential of −0.15 V, and the corresponding calibration curve (inset). (**C**) Amperometric responses upon successive addition of 15 µM NADH, 500 µM uric acid (UA), 100 µM H_2_O_2_, 500 µM dopamine (DA), 100 µM NaCl, 100 µM KCl, 100 µM CaCl_2_, 100 µM MgCl_2_ and 500 µM ascorbic acid (AA), with operational potentials of −0.1 V and −0.15 V in the same conditions as in B (reproduced from [212]).

Besides glucose biosensors, series of other OMC-based enzymatic biosensing devices have been developed for various analytes such as alcohol (using alcohol dehydrogenase, ADH) [194,211,212], catechol, hydroquinone and phenol derivatives (mainly with tyrosinase, TYR) [210,239,240,245,246], or some pesticides (using organophosphorus hydrolase, OPH) [243,244], among others (Table 3). In all cases, similar strategies as above have been applied, using OMC as a support for the enzyme and attempting to keep it durably on the material with polymeric additives (see an illustration on Figure 10A for a TYR-OMC system [246]), along with the possibility to add noble metal NPs to improve the biosensor response [239,240]. The good mechanical stability of the widely open carbonaceous framework and its large specific surface area are responsible for the performance of the bioelectrode, which is reported to be significantly better than analogous devices based on carbon nanotubes instead of OMC (as illustrated on Figure 10B for catechol detection). The biosensor was also applied to tyramine determination in food products [246]. Second generation biosensors have been also developed and an illustration is given for NADH detection at a nanobiocomposite layer made of ADH enzyme, NAD^+^ cofactor, Meldola’s blue mediator, graphitized mesoporous carbon and chitosan as binder (Figure 11A). It shows a fast amperometric response (5 s), excellent sensitivity (10.36 nA∙µM^−1^), and wide linear range (10–410 µM) toward NADH (Figure 11B) and without any other interference signals of common coexisting species (Figure 11C) [212]. It can be applied as ethanol biosensor exhibiting a low detection limit (80 µM) and excellent long-term stability (40 days). Direct electron transfer has been claimed for Horseradish peroxidase (HRP) on OMC modified electrode [237]. When associated to methylene blue mediator, the OMC-HRP system was applied to H_2_O_2_ sensing [234]. HRP was also used to expedite the generation of ZnS quantum dots in OMC and the resulting materials was employed as a sensitive electrochemiluminescence biosensor for glyphosate, based on the inhibition of the activity of HRP by the pesticide [236]. Various organophosphorus pesticides have been determined using OMC bearing OPH enzymes [243,244] or magnetite modified OMC bearing acetylcholinesterase [242]. Copper modified OMC was used as a support for laccase and, after deposition onto a gold electrode; the resulting biosensor was sensitive to catechol [209]. Finally, an interesting work reported that microperoxidase-11 showed a better biosensing performance towards H_2_O_2_ detection when immobilized into a ball-flower-like mesoporous carbon than free enzyme [238]. Such improvement was attributed to the favorable size matching between the enzyme and the mesoporous host.

#### 3.3.3. DNA-Modified OMC-Based Biosensors 

**Figure 12:**
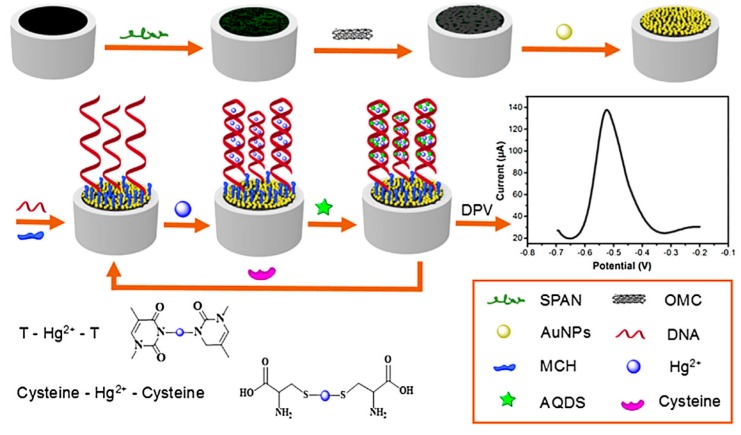
Assembly and detection mechanism of an OMC-DNA-based biosensor for mercury ions (SPAN, self-doped polyaniline; MCH, 6-mercaptohexanol; AQDS, anthraquinone-2,6-disulfonate) (reproduced from [257]).

A Nafion-OMC film deposited onto a carbon ionic liquid paste electrode was applied to the direct electrochemistry of double-stranded DNA (dsDNA), giving well-defined signals recorded by differential pulse voltammetry for the oxidation of adenine and guanine residues, which were directly proportional to dsDNA concentration over a wide range (10–600 µg∙mL^−1^) [270]. Then, biosensing platforms integrating OMC and DNA have been developed for highly sensitive detection of metal ions [257,258]. An example is illustrated in Figure 12 for a reusable ultrasensitive Hg^2+^ biosensor (in the pM concentration range [257]). Its principle involves the folding of DNA probes in the presence of Hg^2+^ ions and subsequent intercalation of an equivalent of anthraquinonedisulfonate giving the voltammetric signal, whereas regeneration was simply achieved using cysteine to destroy the hairpin structure. Another example is the impedimetric biosensing of Pb^2+^ ions using OMC-Au NPs and DNAzyme catalytic beacons [258]. This approach has been also applied to the detection of Ag^+^ ions but using this time an ordered mesoporous carbon nitride support for DNA strands [271].

#### 3.3.4. OMC-Based Immunosensors and Aptasensors

**Figure 13:**
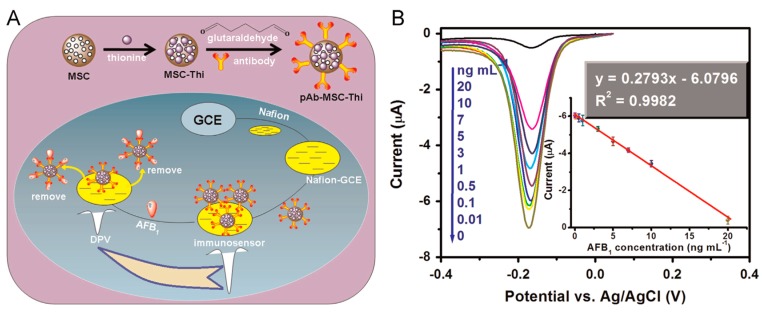
(**A**) Schematic illustration of competitive-type immunosensing strategy, based on target-induced displacement reaction between thionine-decorated mesoporous carbon nanospheres bearing the antibody (pAb-MSC-Thi) and an Nafion-modified glassy carbon electrode (Nafion-GCE) for the electrochemical detection of aflatoxin B_1_ (AFB_1_). (**B**) Differential pulse voltammograms recorded for the immunoassay toward increasing AFB_1_ concentrations (reproduced from [247]).

**Figure 14:**
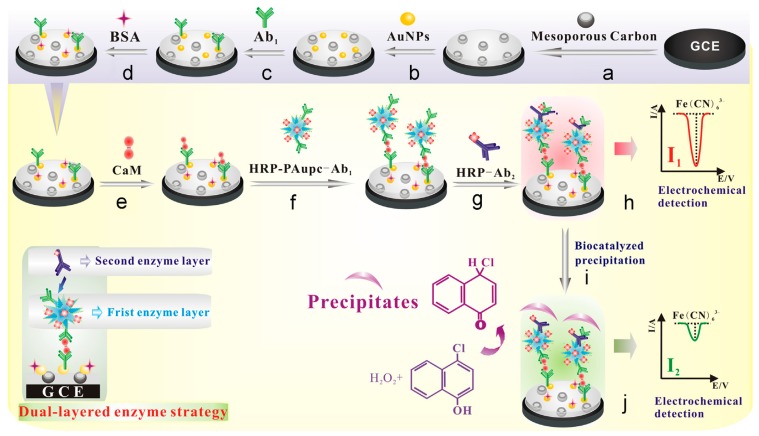
Schematic representation of an electrochemical immunosensor for calmodulin (CaM) based on enhanced biocatalyzed precipitation adopting a dual-layered enzyme strategy using a glassy carbon electrode (GCE) successively covered with mesoporous carbon, gold nanoparticles (AuNPs), Ab_1_ antibody, bovine serum albumin (BSA), the CaM target, horseradish peroxidase and Ab_1_ antigen covalently bound to poly(acrylic acid)-functionalized Au popcorn (HRP-PAupc-Ab_1_) and horseradish peroxidase-secondary antobody (HRP-Ab_2_) (reproduced from [250]).

**Figure 15:**
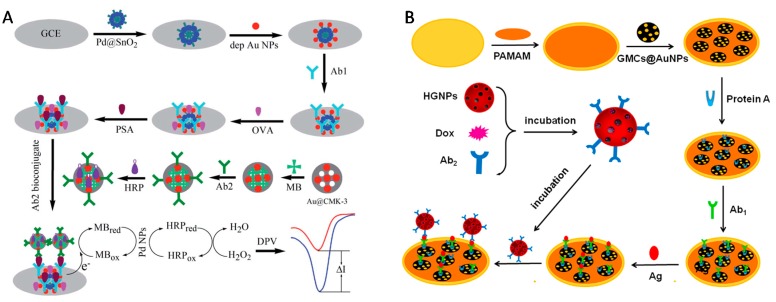
(**A**) Schematic diagram for the stepwise assembly of an electrochemical immunosensor for prostate-specific antigen (PSA) operating in the signal amplification strategy using a glassy carbon electrode (GCE) successively covered with gold nanoparticles (AuNPs) deposited onto flower-like SnO_2_ anchored with palladium NPs (Pd@SnO_2_), Ab_1_ antibody, ovalbumin (OVA), the PSA target, and the bioconjugate in the form of CMK-3 with AuNPs, horseradish peroxidase (HRP) and methylene blue (MB) (reproduced from [256]). (**B**) Fabrication procedure for penicillin binding protein 2 a (PbP2a) immunosensor based on gold electrode covered with polyamidoamine (PAMAM), AuNPs decorated graphitized mesoporous carbon (GMC), protein A and Ab_1_ antibody, the antigen (Ag) and the nanocarriers made of hollow gold nanospheres (HGNPs) incubated with the second antibody (Ab_2_) and doxorubicin (Dox)(reproduced from [254]).

The use of nanomaterials for signal amplification of immunosensors has allowed significant advances in the field of sensitive, portable and easy-to-use devices to detect biomarkers for clinical diagnosis or to monitor organic pollutants in the environment [272]. OMC materials have been exploited in the construction of several immunosensing devices [247,248,249,250,251,252,254,256,273,274] for the detection of specific antigens after binding to immobilized antibodies. The motivation was mainly linked to the large surface area of OMC, ensuring the immobilization of great amounts of antibodies and/or other additional components of the immunosensor (enzymes, mediators, nanoparticles), offering also an electronically conductive matrix likely to enhance the electrochemical transduction. This last point constitutes an advantage with respect to non-conductive mesoporous silica materials that were also largely exploited in electrochemical immunosensors [275]. Various configurations for immunosensor integrating OMC materials have been proposed, as based either on competitive-type immonoassays or signal amplification strategies. An example of the first approach is illustrated in Figure 13 for the detection of aflatoxin B_1_ [247]. It implies antibody attachment to thionine-decorated OMC particles (pAb-MSC-Thi), their accumulation onto a Nafion-modified glassy carbon electrode by electrostatic interactions, and their release in the presence of the target aflatoxin B_1_ (AFB_1_) antigen competing with negatively charged Nafion film for the labelled anti-AFB_1_ on the mesoporous particles, thus resulting in the dissociation of pAb-MSC-Thi from the sensing interface (Figure 13A). This resulted in a decrease in the voltammetric signals of thionine, proportionally to the aflatoxin B_1_ concentration (Figure 13B). Another example applied to the highly sensitive detection of a ubiquitous protein (calmodulin, CaM) is based on a much more sophisticated configuration involving a dual-layered enzyme strategy (Figure 14) and a detection step based on biocatalyzed precipitation inducing a signal decrease proportional to the amount of accumulated CaM (and thus to its concentration); it has been exploited for CaM analysis in cancer cells [250]. The second immunosensing strategy relies on signal amplification. In that case, OMC nanoparticles hosting the enzyme, mediator and antibody, act as nanolabels that are likely to bind to a modified electrode surface containing a second antibody in the presence of the target analyte (the antigen, in a sandwich configuration between the two complementary antibodies) and this leads to an amplification of the bioelectrochemical response proportional to the amount of the accumulated nanolabels and thus to the target antigen concentration [256,273]. An illustration of immunosensor for prostate-specific antigen (PSA) is given in Figure 15A. Metal nanoparticles or nanocarbons (graphene, CNTs) can be added to the device in the goal to improve the electrochemical transduction [248,249,273]. Sometimes, the OMC material is used to prepare the underlying antibody-modified electrode and other types of nanolabels serve for the antigen-antibody binding (see example on Figure 15B) [254].

Finally, two examples of aptasensors (one for detection of human prostate-specific antigen [255] and the other for determination of chlorpyrifos pesticide residues in vegetables and fruits [253]) have been reported, exploiting the high specific surface area and conductivity of OMC for effective signal amplification of the aptamer-target recognition reaction.

## 4. Conclusions

Ordered mesoporous carbon materials are attractive and increasingly used electrode modifiers to design electrochemical (bio)sensors. Like other mesoporous materials, OMCs are characterized by an extremely-open rigid structure and very high specific surface areas that are particularly suited to the adsorption/immobilization of large amounts of reagents while keeping fast mass transport rates in the functionalized materials. But the main interests of OMCs, notably with respect to the widely-used mesoporous silica materials, are their good conductivity (making them real electrode substrates) and their intrinsic electrocatalytic properties. The outstanding performance of OMCs as electrode material for electrochemical sensing and biosensing can be ascribed to the existence of significant edge-plane-like sites and oxygen-rich groups (inducing fast electron transfer kinetics as well as surface reactivity) and the unique regular nanostructure (advantageous for efficient diffusion and transport of reactants and byproducts involved in the application of electrochemical sensors). The main detection schemes are:

- preconcentration electroanalysis (via open-circuit accumulation and subsequent voltammetric detection or electrochemical preconcentration and stripping);
- electrocatalytic detections based on either molecular/organometallic/polymeric mediator species deposited onto the OMC surface or catalytic nanoparticles (metals, metal oxides or sulfides) supported on the OMC host;
- electrochemical biosensors involving bioelectrocatalytic detection mechanisms (based on small redox proteins or enzymes embedded into/onto OMC) and immunosensors or aptasensors.
  Some other sensors (potentiometric, electrochemiluminescent, impedimetric) are also reported.

The main recent trends concern a diversification of the OMC materials used for electrochemical sensing, with significant efforts in using graphitized or partially graphitized OMCs (with enhanced conductivity), nitrogen-doped OMCs (exhibiting improved electrocatalytic properties thanks to a higher number of defect/catalytic sites), or OMC materials characterized by hierarchical and/or multimodal mesostructures (combining optimized hosting features with accelerated mass transport issues). The type of target analytes and nature of reagents used to modify OMC have also expanded. Finally, most applications were based on particulate OMC films (alone or with a polymeric binder), except for a micro glucose sensor based on direct prototyping mesoporous carbon electrode (but the porous carbon layer was not really ordered), and no continuous mesoporous thin film-based electrochemical sensors have been developed to date, even if self-assembly synthesis procedures are established for this kind of materials.

## Figures and Tables

**Table 1:** Electrochemical sensors based on electrodes prepared from templated ordered porous carbon materials (unmodified).

Analyte ^a^		Type of Porous Materials ^b^	Electrode Configuration ^c^	Detection Method		Analytical Performance		Reference
				Procedure ^d^	Technique ^e^	Concentration Range	Det. Limit	
Ascorbic acid Dopamine Uric acid		CMK-1 (KIT-6/PF resin)	GCE/(OMC + Nafion) film	Direct det./EC	DPV	4 × 10^−5^–8 × 10^−4^ M 1 × 10^−6^–9 × 10^−5^ M 5 × 10^−6^–8 × 10^−5^ M	2 × 10^−5^ M 5 × 10^−7^ M 4 × 10^−5^ M	[53]
Ascorbic acid Dopamine Uric acid		Mesoporous carbon nanofiber	PGE/OMC film	Direct det./EC	DPV	1 × 10^−4^–1 × 10^−2^ M 5 × 10^−8^–3 × 10^−5^ M 5 × 10^−7^–1.2 × 10^−4^ M	5 × 10^−5^ M 2 × 10^−8^ M 2 × 10^−7^ M	[54]
Ascorbic acid Dopamine Uric acid		Mesoporous carbon nanofiber	GCE/OMC film	Direct det./EC	A	2 × 10^−8^–2.25 × 10^−3^ M 1 × 10^−7^–7.85 × 10^−4^ M 2 × 10^−8^–5.25 × 10^−5^ M	1.6 × 10^−8^ M 1.6 × 10^−8^ M 1.3 × 10^−8^ M	[55]
Ascorbic acid Dopamine Uric acid		CMK-3 (SBA-15/Furfuryl alcohol)	GCE/OMC film	Direct det./EC	DPV	8 × 10^−5^–1.4 × 10^−3^ M 4 × 10^−7^–6 × 10^−5^ M 1 × 10^−5^–7 × 10^−5^ M	1.4 × 10^−5^ M 2.8 × 10^−7^ M 1.6 × 10^−6^ M	[56]
Bisphenol A		CMK-3 (SBA-15/sucrose)	IL-CPE + OMC	Acc. (3 min)—det.	LSV	2 × 10^−7^–1.5 × 10^−4^ M	5 × 10^−8^ M	[57]
Calcium dobesilate		CMK-3 (SBA-15/sucrose)	PGE/OMC film	Direct det./EC	CV	1 × 10^−7^–1.3 × 10^−3^ M	4.0 × 10^−8^ M	[58]
Capsaicin		Mesoporous cellular foam	CPE + OMC	Acc. (1 min)—det.	DPV	7.6 × 10^−7^–1.16 × 10^−5^ M	8 × 10^−8^ M	[59]
Carbendazim		CMK-3 (SBA-15/sucrose)	IL-CPE + OMC	Acc. (3 min)—det.	DPV	1.25–800 µg/L	0.5 µg/L	[60]
Carvedilol		CMK-1 (MCM-48/sucrose)	GCE/OMC film	Direct det.	DPV	1 × 10^−7^–2.3 × 10^−5^ M	3.4 × 10^−8^ M	[61]
Catechol Hydroquinone		CMK-3 (SBA-15/sucrose)	GCE/(OMC + Nafion) film	Acc. (4 min)—det.	LSV	5 × 10^−7^–3.5 × 10^−5^ M 1 × 10^−6^–3 × 10^−5^ M	1 × 10^−7^ M 1 × 10^−7^ M	[62]
Catechol Hydroquinone		Mesoporous carbon (SBA-15/BMIMPF_6_)	GCE/(OMC + IL) film	Direct det.	DPV	1 × 10^−7^–5 × 10^−5^ M 1 × 10^−7^–5 × 10^−5^ M	6 × 10^−8^ M 5 × 10^−8^ M	[63]
Chloramphenicol		CMK-1 (KIT-6/PF resin)	GCE/OMC/Nafion film	Acc. (4 min)—det.	LSV	5.0 × 10^−7^–6.0 × 10^−5^ M	8.5 × 10^−9^ M	[64]
Chlorogenic acid		DMC (nanosilica/sucrose)	IL-CPE + OMC	Acc. (2 min)—det.	SWV	2 × 10^−8^–2.5 × 10^−6^ M	1 × 10^−8^ M	[65]
Cd^II^ Pb^II^		Mesoporous graphene framework	GCE/(MGF + Nafion) film	Acc. (6 min at −1.2 V)—det.	DPASV	2–70 µg/L 0.5–110 µg/L	0.5 µg/L 0.1 µg/L	[66]
Cu^II^ Pb^II^		CMK-3 (SBA-15/sucrose)	GCE/(OMC + PANI) film	Acc. (2.5 min)—det.	ASV	1 × 10^−8^–1 × 10^−6^ M 2 × 10^−8^–1 × 10^−6^ M	6 × 10^−9^ M 4 × 10^−9^ M	[67]
l-cysteine		CMK-3 (SBA-15/sucrose)	GCE/(OMC + Nafion) film	Direct det./EC	CV	1.8 × 10^−6^–2.5 × 10^−3^ M	2.0 × 10^−9^ M	[25]
l-cysteine Glutathione		CMK-3 (SBA-15/sucrose)	GCE/(OMC + Nafion) film	Direct det./EC	A	3 × 10^−6^–1.3 × 10^−4^ M up to 3 × 10^−3^ M	1 × 10^−8^ M 9 × 10^−8^ M	[68]
DNA bases	purine(G,A)	Mesoporous carbon fibers	GCE/(MCFs + chitosan) film	Direct det./EC	DPV	2.5 × 10^−6^–2.0 × 10^−5^ M 2.5 × 10^−5^–0.9 × 10^−3^ M	4.8 × 10^−7^ M 2.4 × 10^−5^ M	[69]
	pyrimidine (T,C)							
Dopamine		CMK-3 (SBA-15/sucrose)	GCE/OMC film	Direct det./EC	CV	4 × 10^−5^–1 × 10^−3^ M	-	[23]
Epinephrine		CMK-3 (SBA-15/sucrose)	GCE/(OMC + Nafion) film	Direct det./EC	A	1 × 10^−7^–1.2 × 10^−3^ M	3.5 × 10^−8^ M	[70]
Epinephrine		Mesoporous carbon foam	GCE/(MCF + Salep) film	Direct det./EC	DPV	1 × 10^−7^–1.2 × 10^−6^ M	4.0 × 10^−8^ M	[71]
Estrogens		CMK-3 (SBA-15/sucrose)	GPE/(OMC + graphene) film	Acc. (4 min)—det.	SWV	5.0 × 10^−9^–2.0 × 10^−6^ M	2.0 × 10^−9^ M	[72]
Folic acid		CMK-3 (SBA-15/sucrose)	GCE/OMC film	Acc. (10 s)—det.	LSV	5.0 × 10^−10^–1.0 × 10^−7^ M	6.0 × 10^−11^ M	[73]
Glucose		CMK-3 (SBA-15/sucrose)	GCE/OMC film	Direct det./EC	A	5 × 10^−4^–5 × 10^−3^ M	2 × 10^−5^ M	[74]
Hydroquinone		CMK-3 (SBA-15/sucrose)	GCE/OMC film	Direct det./EC	DPV	1.0 × 10^−7^–5.0 × 10^−3^ M	3.14 × 10^−8^ M	[45]
Hydroquinone Catechol Resorcinol		CMK-3 (SBA-15/sucrose)	GCE/OMC film	Direct det./EC	A	1 × 10^−5^–2 × 10^−4^ M 1 × 10^−5^–3 × 10^−4^ M 1 × 10^−5^–1.2 × 10^−4^ M	7.6 × 10^−8^ M 1.0 × 10^−7^ M 9.0 × 10^−8^ M	[75]
Isoniazid		CMK-3 (SBA-15/sucrose)	GCE/(OMC + Nafion) film	Direct det./EC	A	1.0 × 10^−7^–3.7 × 10^−4^ M	8.4 × 10^−8^ M	[76]
Melamine		CMK-3 (SBA-15/sucrose)	GCE/(OMC + Nafion) film	Direct det./EC	DPV	5 × 10^−8^–7 × 10^−6^ M	2.4 × 10^−9^ M	[77]
Methyl parathion		CMK-3 (SBA-15/sucrose)	GCE/OMC film	Acc. (5 min)—det.	LSV	9 × 10^−8^–6.1 × 10^−5^ M	7.6 × 10^−9^ M	[78]
Morphine		CMK-3 (SBA-15/sucrose)	GCE/OMC film	Acc. (5 min)—det.	CV	1 × 10^−7^–2 × 10^−5^ M	1 × 10^−8^ M	[79]
Morphine		CMK-3 (SBA-15/sucrose)	GCE/OMC film	Direct det./EC	A	2 × 10^−7^–1.98 × 10^−4^ M	3 × 10^−8^ M	[80]
NADH		CMK-3 (SBA-15/sucrose)	GCE/OMC film	Direct det./EC	A	5 × 10^−6^–9 × 10^−4^ M	1.6 × 10^−6^ M	[81]
NADH		CMK-3 (SBA-15/sucrose)	GCE/(OMC + Nafion) film	Direct det./EC	A	2 × 10^−6^–1.1 × 10^−3^ M	1.0 × 10^−6^ M	[82]
NADH		CMK-1 (KIT-6/sucrose)	GCE/(OMC + Nafion) film	Direct det./EC	A	3.0 × 10^−6^–1.4 × 10^−3^ M	1.0 × 10^−6^ M	[83]
Nitrite		HONC (SBA-15/Furfuryl alcohol)	GCE/OMC film	Acc. (2 min)—det.	DPV	7.0 × 10^−6^–1.6 × 10^−3^ M	-	[84]
Nitroaromatic (TNT)		CMK-3 (SBA-15/sucrose)	GCE/(OMC + Nafion) film	Acc. (2 min)—det.	AdsSV	1–50 ppb	0.2 ppb	[85]
Nitroaromatic (TNT, TNB, DNT, DNB)		CMK-3 (SBA-15/sucrose)	CDE/OMC/Nafion film	Direct det./EC	CE-A	8.4–5.0 µg/L	3–4.7 µg/L	[86]
Nitrobenzene		Bi-modal MMPCM	GCE/OMC film	Acc.—det.	LSV	2 × 10^−7^–4 × 10^−5^ M	8 × 10^−9^ M	[87]
Nitrophenols	o-NP	CMK-3 (SBA-15/sucrose)	GCE/(OMC + Nafion) film	Direct det./EC	DPV	5 × 10^−7^–1.0 × 10^−4^ M 1 × 10^−6^–1.0 × 10^−4^ M 2 × 10^−6^–9.0 × 10^−5^ M	8 × 10^−8^ M 6 × 10^−8^ M 1 × 10^−7^ M	[88]
	m-NP							
	p-NP							
Nitroxoline		CMK-3 (SBA-15/sucrose)	CPE + OMC	Acc. (4 min)—det.	SW-AdsSV	1.0 × 10^−11^–1.0 × 10^−7^ M	3.0 × 10^−12^ M	[89]
Paraoxon parathion methyl parathion		CMK-3 (SBA-15/sucrose)	GCE/(OMC + Nafion) film	Acc. (6 min at −0.6 V)—det.	DPV	1.0 × 10^−8^–2.0 × 10^−5^ M 1.5 × 10^−8^–1.0 × 10^−5^ M 1.0 × 10^−8^–1.0 × 10^−5^ M	1.9 × 10^−9^ M 3.4 × 10^−9^ M 2.1 × 10^−9^ M	[90]
Pb^II^		CMK-3 (SBA-15/sucrose)	GCE/(OMC + Nafion) film	Acc. (5 min)—det.	ASDPV	2 × 10^−8^–2 × 10^−6^ M	4.6 × 10^−9^ M	[91]
Pb^II^		CMK-3 (SBA-15/sucrose)	CILE/(OMC + ENIM-BF_4_ + chitosan) film	Acc. (200 s)—det.	ASDPV	5 × 10^−8^–1.4 × 10^−6^ M	2.5 × 10^−8^ M	[92]
Poly-phenols	1,4-DHB	Graphitized mesoporous carbon	GCE/OMC film	Direct det.	DPV	4.0 × 10^−5^–2.5 × 10^−4^ M 2.5 × 10^−5^–2.0 × 10^−4^ M 2.5 × 10^−5^–2.0 × 10^−4^ M	9.1 × 10^−7^ M 1.31 × 10^−6^ M 6.7 × 10^−7^ M	[93]
	1,2-DHB							
	1,3-DHB							
Prednisolone		OMC	GCE/OMC film	Direct det./EC	SWV	6 × 10^−8^–4.0 × 10^−5^ M	5.7 × 10^−8^ M	[94]
Ractopamine		CMK-3 (SBA-15/sucrose)	GCE/OMC film	Direct det./EC	DPV	8.5 × 10^−8^–8.0 × 10^−6^ M	6 × 10^−8^ M	[95]
Riboflavin		CMK-3 (SBA-15/sucrose)	GCE/OMC film	Acc.—det.	CV	4.0 × 10^−7^–1.0 × 10^−6^ M	2 × 10^−8^ M	[96]
Rutin		DMC	IL-CPE + OMC	Acc. (7 min)—det.	SWV	8 × 10^−9^–4.0 × 10^−6^ M	1.17 × 10^−9^ M	[97]
Sudan I		CMK-3 (SBA-15/sucrose)	GCE/OMC film	Acc.—det.	ASDPV	4.0 × 10^−7^–6.6 × 10^−5^ M	2.44 × 10^−9^ M	[98]
Tirapazamine		CMK-3 (SBA-15/sucrose)	PGE/OMC film	Acc.—det.	DPV	5 × 10^−11^–1.5 × 10^−5^ M	2.0 × 10^−11^ M	[99]
Triclosan		CMK-3 (SBA-15/sucrose)	SPCE/(OMC + chitosan) film	Direct det.	SWV	0.8–40 µg/L	0.24 µg/L	[43]
l-tyrosine		CMK-3 (SBA-15/Furfuryl alcohol)	GCE/OMC film	Direct det./EC	DPV	1.5 × 10^−5^–9 × 10^−4^ M	1.0 × 10^−5^ M	[100]
Uric acid		CMK-3 (SBA-15/sucrose)	GCE/OMC film	Direct det./EC	CV	7 × 10^−6^–1.5 × 10^−4^ M	2.0 × 10^−6^ M	[101]
Uric acid		CMK-3 (SBA-15/sucrose)	PGE/OMC film	Direct det./EC	A	1.0 × 10^−6^–1.0 × 10^−4^ M	4.0 × 10^−7^ M	[102]
Xanthine Hypoxanthine Uric acid		Graphitized mesoporous carbon	GCE/OMC film	Direct det./EC	DPV	2 × 10^−5^–2.4 × 10^−4^ M 2 × 10^−5^–3.2 × 10^−4^ M 2 × 10^−5^–4.0 × 10^−4^ M	3.51 × 10^−7^ M 3.88 × 10^−7^ M 1.10 × 10^−7^ M	[44]

^a^ Abbreviations: NADH, nicotinamide adenine dinucleotide (reduced form); TNT, 2,4,6-trinitrotuluene; TNB, 1,3,5-trinitrobenzene; DNT, 2,4-dinitrotuluene; DNB, 1,3-dinitrobenzene; NP, nitrophenol; DHB, dihydroxy benzene; DNA (deoxyribonucleic acid) bases (A, adenine; G, guanine; C,cytosine; T, thymine). ^b^ Abbreviations: CMK, carbon mesostructures at KAIST; KIT, Korean Institute of Technology; PF resin, phenol formaldehyde resin; SBA, Santa Barbara Amorphous; MCM, Mobil Composition of Matter; BMIMPF6, ionic liquid of 1-octyl-3-methylimidazolium bromide; DMC, highly defective mesoporous carbon; HONC, hemi-ordered nanoporous carbon; MMPCM, micro-/meso-porous carbon material. ^c^ Abbreviations: GCE, glassy carbon electrode; OMC, ordered mesoporous carbon (general term to name the porous carbon material, even if modified, as detailed in the preceeding column); PGE, pyrolytic graphite electrode; IL, ionic liquid; CPE, carbon paste electrode; MGF, mesoporous graphene framework; PANI, polyaniline; MCF+Salep, mesoporous carbon foam dispersed in Salep; GPE, graphite paste electrode; CDE, carbon disk electrode; CILE, carbon ionic liquid electrode; ENIM-BF4, 1-ethyl-3-methylimidazolium tetrafluoroborate ionic liquid; MCFs, mesoporous carbon fibers; SPCE, scree-printed carbon electrode. ^d^ Abbreviations: det., detection; EC, electrocatalysis; acc., accumulation. ^e^ Abbreviations: DPV, differential pulse voltammetry; A, amperometry; LSV, linear sweep voltammetry; CV, cyclic voltammetry; SWV, square wave voltammetry; DPASV, differential pulse anodic stripping voltammetry; ASV, anodic stripping voltammetry; AdsSV, adsorptive stripping voltammetry; CE-A, capillary electrophoresis with amperometric detection; SW-AdsSV, square wave adsorptive stripping voltammetry; ASDPV, anodic stripping differential pulse voltammetry.

**Table 2:** Electrochemical sensors based on electrodes prepared from functionalized templated ordered porous carbon materials.

Analyte ^a^	Porous Materials		Electrode Configuration ^d^	Detection Method		Analytical Performance		Reference
	Type ^b^	Modifier ^c^		Procedure ^e^	Technique ^f^	Concentration Range	Det. Limit	
Acetylcholine	CMK-3 (SBA-15/sucrose)	Ni-Al LDH	GCE/OMC/LDH film	Mediated EC	A	2 × 10^−6^–4.92 × 10^−3^ M	4.2 × 10^−8^ M	[158]
Ag^+^	Macroporous carbon	Ag^+^ ionophores	OMC/membrane	Direct det.	P	10^−10^–10^−11^ M	3.8 × 10^−11^ M	[187]
Ascorbic acid	CMK-3 (SBA-15/sucrose)	Ferrocene-COOH	GCE/(OMC + Nafion) film	Mediated EC	A	5 × 10^−5^–3.5 × 10^−4^ M	9 × 10^−6^ M	[112]
Ascorbic acid Dopamine Uric acid	N-PCNPs (ZIF-8/Zn(Ac)_2_/methylimidazole)	N (nitrogen)	GCE/OMC film	Supported EC	DPV	8 × 10^−5^–2 × 10^−3^ M 5 × 10^−7^–3 × 10^−5^ M 4 × 10^−6^–5 × 10^−5^ M	7.4 × 10^−7^ M 1.1 × 10^−8^ M 2.1 × 10^−8^ M	[107]
Ascorbic acid Dopamine Uric acid	N-doped OMC (SBA-15/poly(ethylenediamine))	N (nitrogen)	GCE/(OMC + Nafion) film	Direct det./EC	SWV	1 × 10^−6^–7 × 10^−4^ M 1 × 10^−9^–3 × 10^−5^ M 1 × 10^−8^–8 × 10^−5^ M	1 × 10^−8^ M 1 × 10^−9^ M 1 × 10^−8^ M	[108]
Ascorbic acid Dopamine Uric acid	N-doped OMC (3-amino phenol/formaldehyde resin)	N (nitrogen)	GCE/OMC film	Direct det./EC	SWV	1.0 × 10^−6^–1.2 × 10^−4^ M 5 × 10^−8^–1.45 × 10^−5^ M 2.0 × 10^−6^–3.0 × 10^−5^ M	1.0 × 10^−7^ M 2.0 × 10^−8^ M 1.4 × 10^−7^ M	[109]
6-Benzylaminopurine	CMK-3 (SBA-15/sucrose)	Platinum NPs	GCE/(OMC + Nafion) film	Supported EC	A	5 × 10^−8^–2.4 × 10^−5^ M	5 × 10^−9^ M	[139]
Bromate Iodate Nitrite Hydrogen peroxide	CMK-3 (SBA-15/sucrose)	P_2_Mo_18_	GCE/(OMC + PVA) film	Mediated EC	A	2.77 × 10^−6^–4 × 10^−3^ M 1.13 × 10^−6^–6.2 × 10^−3^ M 5.34 × 10^−6^–2.4 × 10^−2^ M 1.6 × 10^−4^–4.4 × 10^−2^ M	9.22 × 10^−7^ M 3.77 × 10^−7^ M 1.78 × 10^−6^ M 5.34 × 10^−5^ M	[24]
Captopril	OMC	CuHCF	GCE/OMC/CuHCF film	Direct det./EC	CV	1.0 × 10^−5^–2.7 × 10^−3^ M	1.2 × 10^−6^ M	[122]
Catechol	N-doped OMC (SBA-15/ethyl violet)	N (nitrogen)	GCE/OMC film	Direct det./EC	DPV	6 × 10^−6^–7 × 10^−5^ M	0.9 × 10^−6^ M	[49]
Cd^II^	OMC	Sn-Pb NPs	GCE/OMC film	Acc.—det.	SWASV	1–140 µg∙L^−1^	0.36 µg∙L^−1^	[156]
Ciprofloxacin	CMK-3 (SBA-15/sucrose)	CTAB (0.1 mM)	CPE + OMC (15%)	Acc. (4 min)—det.	LS-AdsSV	5.0 × 10^−9^–2.0 × 10^−5^ M	1.5 × 10^−9^ M	[133]
Citrinin	CMK-3 (SBA-15/sucrose)	Gold NPs	AuE/OMC/MIP film	Direct det.	EQCM	6.0 × 10^−9^–2.0 × 10^−7^ M	1.8 × 10^−9^ M	[140]
Cl^−^	CIM carbon	IL-PVC	OMC-IL-PVC membrane	Direct detection	P	3.16 × 10^−4^–1 × 10^−1^ M	-	[130]
Dimetridazole	CMK-3 (SBA-15/sucrose)	Gold NPs/MIP	GCE/GO/OMC/MIP film	Acc. (3 min)—det.	DPV	2.0 × 10^−9^–2.5 × 10^−7^ M	5.0 × 10^−10^ M	[128]
l-Dopa	Large mesoporous C	CoHCF	GCE/OMC film	Mediated EC	A	1.0 × 10^−7^–1.9 × 10^−3^ M	1.7 × 10^−8^ M	[123]
Dopamine	CMK-3 (SBA-15/sucrose)	unmodified -COOH -NH_2_	GCE/OMC film	Direct det.	DPV	5 × 10^−8^–1.0 × 10^−6^ M 2.0 × 10^−7^–1.96 × 10^−6^ M 6 × 10^−7^–1.26 × 10^−5^ M	4.5 × 10^−9^ M 4.4 × 10^−8^ M 3.3 × 10^−7^ M	[50]
Dopamine	OMC	Ru(bpy)_3_^2+^	GCE/(OMC + Nafion) film	Direct det.	ECL	5.0 × 10^−9^–5.0 × 10^−4^ M	1.7 × 10^−9^ M	[114]
Dopamine, Uric acid	CMK-3 (SBA-15/sucrose)	-COOH, IL	OMC + IL mixture rubbed onto GCE	Acc.—det.	DPV	1 × 10^−7^–5 × 10^−4^ M 1 × 10^−7^–1 × 10^−4^ M	4.1 × 10^−9^ M 2.5 × 10^−9^ M	[131]
Estradiol	CMK-3 (SBA-15/sucrose)	Poly(l-proline)	GCE/(OMC + l-proline) film	Mediated EC	SWV	1.0 × 10^−8^–2.0 × 10^−6^ M	5.0 × 10^−9^ M	[181]
Ethanol, Glycine	CMK-3 (SBA-15/sucrose)	Ni(OH)_2_ NPs	GCE/(OMC + Nafion) film	Mediated EC	A	up to 8.0 × 10^−2^ M up to 3.2 × 10^−3^ M	4.77 × 10^−6^ M 2.6 × 10^−6^ M	[159]
Glucose	CMK-3 (SBA-15/sucrose)	Platinum NPs	GCE/(OMC + Nafion) film	Supported EC	A	5 × 10^−6^–7.5 × 10^−3^ M	3 × 10^−6^ M	[141]
Glucose	CMK-3 (SBA-15/sucrose)	NiO	GCE/(OMC + Nafion) film	Supported EC	A	2 × 10^−6^–1 × 10^−3^ M	6.5 × 10^−7^ M	[160]
Glucose	Onion-like OMC	PtPd NPs	GCE/(OMC + Nafion) film	Supported EC	A	1.5 × 10^−3^–1.2 × 10^−2^ M	-	[157]
Glucose	CMK-3 (SBA-15/Furfuryl alcohol)	CuO NPs	GCE/(OMC + Nafion) film	Supported EC	A	1 × 10^−5^–1 × 10^−3^ M	-	[161]
Glucose	OMC (colloidal SiO_2_/F127/phenolic resin)	CuO NPs	GCE/OMC film	Supported EC	A	4 × 10^−7^–7.3 × 10^−3^ M	1 × 10^−7^ M	[162]
Glucose	Carbon aerogel (resorcinol/formaldehyde)	Iron/iron oxide	CPE + OMC	Supported EC	A	1 × 10^−3^–5.0 × 10^−2^ M	-	[163]
Glucose	IFMC	Palladium NPs	GCE/OMC/Nafion film	Supported EC	A	1 × 10^−3^–5.5 × 10^−2^ M	2 × 10^−4^ M	[142]
Glucose	OMC	Co_3_O_4_ nanocrystals	GCE/(OMC + Nafion) film	Supported EC	A	1 × 10^−5^–0.8 × 10^−3^ M	1 × 10^−6^ M	[164]
Glutathione	CMK-3 (SBA-15/sucrose)	Co oxide (Co_3_O_4_)	GCE/(OMC + Nafion) film	Mediated EC	A	4 × 10^−6^–2.8 × 10^−5^ M	1.4 × 10^−10^ M	[165]
Guanine Adenine	CMK-3 (SBA-15/sucrose)	CePW	GCE/OMC film	Mediated EC	CV	4 × 10^−6^–1.9 × 10^−3^ M 4.0 × 10^−6^–7.0 × 10^−4^ M	5.7 × 10^−9^ M 7.45 × 10^−8^ M	[182]
H^+^ (pH sensor)	OMC	RuO_2_ film	OMC-SPE	Direct detection	P	1 × 10^−10^–1 × 10^−4^ M	-	[166]
Hg^II^	Mesoporous C nanofiber	BIM ligand	GCE/(OMC+BIM+Nafion) film	Acc. (15 min)—det.	DPASV	5 × 10^−9^–5 × 10^−7^ M	3 × 10^−10^ M	[127]
Hydrazine	CMK-3 (SBA-15/sucrose)	PDDA—Pt NPs	GCE/(OMC + Nafion) film	Supported EC	A	5 × 10^−6^–1.35 × 10^−4^ M	1.7 × 10^−7^ M	[143]
Hydrazine	CMK-3 (SBA-15/sucrose)	CeHCF	GCE/(OMC + Nafion) film	Mediated EC	A	1 × 10^−6^–1.63 × 10^−4^ M	1 × 10^−7^ M	[124]
Hydrazine	OMC	CeO_2_ NPs	GCE/OMC/Nafion film	Supported EC	A	4.0 × 10^−8^–1.92 × 10^−4^ M	1.2 × 10^−8^ M	[167]
Hydrazine	OMC	Curcumin	GCE/OMC film	Direct det./EC	A	1.25 × 10^−5^–2.25 × 10^−4^ M	3.9 × 10^−7^ M	[120]
Hydrazine	CMK-1 (MCM-48/sucrose)	SDS—Pd NPs	GCE/(OMC + Nafion) film	Supported EC	A	3 × 10^−6^–1 × 10^−3^ M	1.16 × 10^−6^ M	[134]
Hydrazine Hydrogen peroxide Nitrobenzene	CMK-1 (MCM-48/sucrose)	Platinum NPs	GCE/(OMC + Nafion) film	Supported EC	A	1 × 10^−5^–8.4 × 10^−4^ M 5 × 10^−6^–5.4 × 10^−3^ M 4 × 10^−6^–6.7 × 10^−4^ M	3.41 × 10^−6^ M 1.09 × 10^−6^ M 3.82 × 10^−6^ M	[144]
Hydrogen peroxide	CMK-3 (SBA-15/sucrose)	Fe oxide (Fe_3_O_4_)	GCE/(OMC + Nafion) film	Mediated EC	A	7 × 10^−6^–4 × 10^−3^ M	3.6 × 10^−8^ M	[168]
Hydrogen peroxide	CMK-3 (SBA-15/sucrose)	Cu_2_S	GCE/(OMC + Nafion) film	Mediated EC	A	1 × 10^−6^–3.03 × 10^−3^ M	2 × 10^−7^ M	[169]
Hydrogen peroxide	CMK-3 (SBA-15/sucrose)	Gold NPs	GCE/OMC film	Supported EC	A	2.0 × 10^−6^–3.92 × 10^−3^ M	4.9 × 10^−7^ M	[145]
Hydrogen peroxide	CMK-3 (SBA-15/sucrose)	Platinum NPs	GCE/OMC-PIL film	Supported EC	A	1.0 × 10^−7^–3.2 × 10^−3^ M	8 × 10^−8^ M	[146]
Hydrogen peroxide	CMK-3 (SBA-15/sucrose)	Palladium NPs	GCE/(OMC + Nafion) film	Supported EC	A	7.5 × 10^−6^–1.0 × 10^−2^ M	1.0 × 10^−6^ M	[147]
Hydrogen peroxide	LMC (CaCO_3_/sucrose)	Co(salen)	GCE/(OMC + Nafion) film	Mediated EC	A	2.0 × 10^−6^–8.9 × 10^−3^ M	8.5 × 10^−7^ M	[117]
Hydrogen peroxide	CMK-3 (SBA-15/sucrose)	MnO_2_	GCE/(OMC + Nafion) film	Supported EC	A	5 × 10^−7^–6 × 10^−4^ M	7.8 × 10^−8^ M	[170]
Hydrogen peroxide	OMC (SBA-15/glucose)	Silver NPs	GCE/OMC film	Supported EC	A	0.1 × 10^−6^–4.1 × 10^−5^ M	5.0 × 10^−8^ M	[148]
Hydrogen peroxide	CMK-5 (SBA-15/furfuryl alcohol)	Fe-PTPY (grafted or adsorbed)	GCE/OMC film	Mediated EC	A	1 × 10^−5^–1.3 × 10^−2^ M	2 × 10^−6^ M	[118]
Hydrogen peroxide	OMC	pFeMOF	GCE/(OMC + Nafion) film	Supported EC	A	5 × 10^−7^–7.05 × 10^−5^ M	4.5 × 10^−7^ M	[116]
Hydrogen peroxide	OMC (colloidal SiO_2_/F127/phenolic resin)	Hemin	GCE/(OMC + Nafion + hemin) film	Direct det./EC	A	2.0 × 10^−6^–2.5 × 10^−4^ M	3 × 10^−7^ M	[115]
Hydrogen peroxide Ascorbic acid	CMK-3 (SBA-15/sucrose)	Prussian Blue	GCE/OMC film	Mediated EC	A	4 × 10^−4^–5.6 × 10^−3^ M 1 × 10^−4^–1.4 × 10^−3^ M	1 × 10^−6^ M 2.6 × 10^−7^ M	[125]
Hydrogen peroxide Glucose	CMK-3 (SBA-15/sucrose)	Platinum NPs	GCE/(OMC + Nafion) film	Supported EC	A	2 × 10^−6^–4.21 × 10^−3^ M 5 × 10^−4^–4.5 × 10^−3^ M	1.2 × 10^−6^ M 1.3 × 10^−4^ M	[149]
Hydrogen peroxide Glucose	Macroporous carbon	CuO nanoneedles	GCE/OMC film	Supported EC	A	1.0 × 10^−5^–6.5 × 10^−3^ M 3.5 × 10^−6^–3.0 × 10^−4^ M	2 × 10^−7^ M 2 × 10^−6^ M	[171]
Hydrogen peroxide Hydrazine	Onion-like OMC	Palladium NPs	GCE/(OMC + Nafion) film	Supported EC	A	1.0 × 10^−7^–6.1 × 10^−3^ M 2.0 × 10^−8^–7.1 × 10^−5^ M	7.9 × 10^−8^ M 1.49 × 10^−8^ M	[150]
Hydrogen peroxide NADH Acetaminophenol	CMK-3 (SBA-15/sucrose)	POMs/gold NPs	GCE/Au@POMs/OMC film	Supported EC	A	1 × 10^−6^–2.0 × 10^−5^ M 1 × 10^−6^–1.1 × 10^−4^ M 1 × 10^−6^–5.7 × 10^−5^ M	3.6 × 10^−7^ M 4.1 × 10^−7^ M 2.9 × 10^−7^ M	[119]
Hydrogen peroxide Nitrite	LMC (CaCO_3_/sucrose)	PDDA/gold NPs	GCE/OMC-PDDA-Ag- or Au-NPs/chitosan film	Supported EC	A	2.0 × 10^−5^–9.62 × 10^−3^ M 5 × 10^−6^–7.24 × 10^−3^ M	6.5 × 10^−6^ M 4.2 × 10^−7^ M	[151]
Hydroquinone catechol	N,S dual-doped OMC	N (nitrogen) and S (sulfur)	GCE/OMC film	Direct det./EC	DPV	1 × 10^−6^–1.1 × 10^−4^ M 1 × 10^−6^–1.1 × 10^−4^ M	5.6 × 10^−8^ M 2.1 × 10^−7^ M	[111]
Iodate	OMC (MCM-41/sucrose)	Silver NPs	GCE/OMC film	Supported EC	A	1.5 × 10^−5^–4.43 × 10^−3^ M	3.01 × 10^−6^ M	[152]
Luminol Hydrogen peroxide	CMK-3 (SBA-15/sucrose)	PANI	GCE/OMC/PANI film	Direct det.	ECL	1.0 × 10^−7^–5.0 × 10^−5^ M 1.0 × 10^−7^–1.0 × 10^−5^ M	8.8 × 10^−10^ M	[173]
K^+^	Macroporous carbon	K^+^ ionophore	Ni/OMC/membrane	Direct det.	P	1.0 × 10^−7^–3.7 × 10^−4^ M	10^−6.2^ M	[26]
K^+^	CIM carbon	K^+^ ionophore	Au/(OMC + PVC) film/valinomycine membrane	Direct det.	P	1 × 10^−5^–1 × 10^−1^ M	10^−5.6^ M	[189]
K^+^ Ag^+^	Macroporous carbon	K^+^ ionophore	Ni/OMC/membrane	Direct det.	P	10^−6^–10^−3^ M 10^−10^–10^−8^ M	1.6 × 10^−7^ M 4.0 × 10^−11^ M	[188]
Metolcarb	CMK-3	Prussian Blue	GCE/OMC film	Acc. (5 min)— Mediated EC	LSV	5.0 × 10^−10^–1.0 × 10^−4^ M	9.3 × 10^−11^ M	[186]
2-mercaptoethanol	CMK-3 (SBA-15/sucrose)	DDAB/bi-CoPc	GCE/DDAB/OMC film	Mediated EC	A	2.5 × 10^−6^–1.4 × 10^−4^ M	6 × 10^−7^ M	[126]
NADH	CMK-3 (SBA-15/sucrose)	C_60_	GCE/OMC film	Direct det./EC	A	1.0 × 10^−7^–9.0 × 10^−4^ M	3 × 10^−8^ M	[184]
NADH	CMK-3 (SBA-15/sucrose)	Nile Blue	GCE/OMC film	Mediated EC	A	5.0 × 10^−5^–1.25 × 10^−3^ M	1.2 × 10^−6^ M	[178]
NADH	CMK-3 (SBA-15/sucrose)	Toluidine Blue O	GCE/(OMC + IL) film	Mediated EC	A	1.0 × 10^−6^–1.0 × 10^−3^ M	4 × 10^−7^ M	[177]
NADH	CMK-3 (SBA-15/sucrose)	Polythionine	GCE/OMC film	Mediated EC	A	3.4 × 10^−6^–8.5 × 10^−4^ M	5.1 × 10^−8^ M	[176]
NADH	CMK-3 (SBA-15/sucrose)	Poly(Azure B)	GCE/OMC film	Mediated EC	A	3.0 × 10^−6^–1.0 × 10^−3^ M	1.0 × 10^−7^ M	[175]
NADH	CMK-3 (SBA-15/sucrose)	Poly(neutral red)	GCE/OMC film	Mediated EC	A	up to 1.6 × 10^−3^ M	1.5 × 10^−7^ M	[179]
NADH	CMK-3 (SBA-15/sucrose)	Polycatechol	GCE/OMC film	Mediated EC	A	up to 2.5 × 10^−4^ M	2 × 10^−7^ M	[180]
Nitrobenzene	N-doped OMC (SBA-15/(NH_4_)_2_S_2_O_8_/aniline)	Ag NPs	GCE/(OMC + Nafion) film	Supported EC	DPV	6.6 × 10^−8^–1.1 × 10^−6^ M	6.6 × 10^−9^ M	[153]
Ofloxacin	MCNs	MIP	GCE/MCNs@MIP	Acc.—det.	CV	5 × 10^−7^–1 × 10^−4^ M	8.0 × 10^−8^ M	[129]
Omeprazole	OMC (fructose/F127)	MPTES	CPE + OMC (5%)	Acc. (60 s)—det.	DPV	0.25 × 10^−9^–2.5 × 10^−7^ M	0.04 × 10^−9^ M	[183]
Oxygen (dissolved)	CMK-3 (SBA-15/sucrose)	TTF	GCE/(OMC + chitosan + Nafion) film	Direct det./EC	A	7 × 10^−6^–1.93 × 10^−4^ M	3.9 × 10^−7^ M	[121]
Pb^II^	Oxidized OMC (F127, resorcinol/formaldehyde)	Fe_3_O_4_	Graphite rod/OMC film	Acc. (6 min)—det.	SWASV	0.005–0.445 mg∙L^−1^	1.57 µg∙L^−1^	[106]
Pb^II^ Cd^II^	CMK-3 (SBA-15/sucrose)	Bismuth(III)	CPE + OMC	Acc. (150 s)—det.	SWASV	1–70 µg∙L^−1^	0.08 µg∙L^−1^ 0.07 µg∙L^−1^	[190]
Pb^II^ Cd^II^	HMCS	Bismuth oxide	GCE/(OMC + chitosan) film	Acc. (150 s)—det.	SWASV	3 × 10^−12^–2.1 × 10^−11^ M 3 × 10^−12^–2.1 × 10^−11^ M	1.7 × 10^−12^ M 1.6 × 10^−12^ M	[172]
Pb^II^ Cd^II^	CMK-3 (SBA-15/sucrose)	PANI-MES	GCE/OMC/PANI-MES film	Acc. (150 s)—det.	DPASV	1 × 10^−9^–1.2 × 10^−7^ M	1.6 × 10^−10^ M 2.6 × 10^−10^ M	[174]
Quercetin	OMC	IL-MoS_2_-Pd NPs	GCE/OMC film	Supported EC	LSV	2.0 × 10^−8^–1.0 × 10^−5^ M	8.0 × 10^−9^ M	[132]
Ractopamine	OMC	Electrodeposited Au	GCE/OMC film	Supported EC	DPV	3 × 10^−8^–7.5 × 10^−5^ M	4.4 × 10^−9^ M	[155]
Ractopamine	OMC	Electrodeposited Au NPs	SPCE/OMC/AuNPs/MIM film	Acc. (100 s)—det.	DPV	5 × 10^−11^–1 × 10^−9^ M	4.2 × 10^−11^ M	[154]
Superoxide anion	N-doped HMCS (silica/resorcinol/formaldehyde)	N (nitrogen)	SPCE/OMC film	Direct det./EC	A	2.0 × 10^−5^–4.8 × 10^−4^ M	2.2 × 10^−6^ M	[110]
Theophylline	LMC (CaCO_3_/sucrose)	SWCNT	GCE/(OMC-SWCNT + Nafion) film	Acc. (100 s)—det.	DPV	3 × 10^−7^–3.8 × 10^−5^ M	8 × 10^−8^ M	[185]
Uric acid	CMK-3 (SBA-15/sucrose)	Ferrocene-COOH	GCE/(OMC + Nafion) film	Mediated EC	A	6 × 10^−5^–3.9 × 10^−4^ M	1.8 × 10^−6^ M	[113]

^a^ Abbreviations: NADH, nicotinamide adenine dinucleotide (reduced form). ^b^ Abbreviations: CMK, carbon mesostructures at KAIST; SBA, Santa Barbara Amorphous; N-PCNPs, nitrogen-doped porous carbon nanopolyhedra; ZIF-8, zeolitic imidazolate framework-8; CIM carbon, colloid-imprinted mesoporous carbon; IFMC, ionic liquid derived fibrillated mesoporous carbon; LMC, large mesoporous carbon; MCNs, mesoporous carbon nanoparticles; HMCS, hollow mesoporous carbon spheres. ^c^ Abbreviations: Ni-Al LDH, nickel-aluminium layered double hydroxide; Ferrocene-COOH, ferrocene-carboxylic acid; NPs, nanoparticles; P_2_Mo_18_ , polyoxometalate; CuHCF, copper hexacyanoferrate; CTAB, cetyltrimethylammonium bromide; IL-PVC, ionic liquid poly(vinyl chloride); MIP, molecularly imprinted polymer; CoHCF, cobalt hexacyanoferrate; IL, ionic liquid; CePW, cerium(III) 12-tungstophosphoric acid; BIM, bis(indoyl)methane; PDDA, poly(diallylammonium chloride); CeHCF, cerium hexacyanoferrate; SDS, sodium dodecyl sulfate; Co(salen), [*N*,*N’*-bis(salicylaldehyde) ethylenediimino cobalt(III)]; Fe-PTPY, iron terpyridine complex; pFeMOF, porphyrinic iron metal-organic framework; POMs, polyoxometalates; BMIMPF_6_, ionic liquid of 1-octyl-3-methylimidazolium bromide; PANI, polyaniline; DDAB/bi-CoPc, binuclear cobalt phthalocyanine exchanged on a didodecyldimethylammonium bromide film; C_60_, fullerene; MIP, molecularly imprinted polymer; MPTES, mercaptopropyltriethoxysilane; TTF, tetrathiafulvalene; PANI-MES, 2-mercaptoethanesulfonate-tethered polyaniline; SWCNT, single walled carbon nanotube. ^d^ Abbreviations: GCE, glassy carbon electrode; OMC, ordered mesoporous carbon (general term to name the porous carbon material, even if modified, as detailed in the preceeding columns); LDH, layered double hydroxide; PVA, poly(vinyl alcohol); CuHCF, copper hexacyanoferrate; CPE, carbon paste electrode; AuE, gold electrode; IL-PVC membrane, ionic liquid poly(vinyl chloride) membrane; GO, graphene oxide; MIP, molecularly imprinted polymer; IL, ionic liquid; SPE, screen-printed electrode; BIM, bis(indoyl)methane; PIL, poly(ionic liquid); Au@POMs/OMC, gold nanoparticles and polyoxometalates decorated OMC; PDDA-Ag- or Au-NPs, silver or gold nanoparticles onto poly(diallyldimethylammonium chloride); PANI, polyaniline; PVC, poly(vinyl chloride); DDAB, didodecyldimethylammonium bromide; MCNs@MIP, molecularly imprinted polymer/mesoporous carbon nanoparticles composite; MES, mercaptoethanesulfonate; SPCE, screen-printed carbon electrode; MIM, molecular imprinted membrane; SWCNT, single walled carbon nanotube. ^e^ Abbreviations: EC, electrocatalysis; det., detection; acc., accumulation. ^f^ Abbreviations: A, amperometry; P, potentiometry; DPV, differential pulse voltammetry; SWV, square wave voltammetry; CV, cyclic voltammetry; SWASV, square wave anodic stripping voltammetry; LS-AdsSV, linear sweep adsorptive stripping voltammetry; EQCM, electrochemical quartz crystal microbalance; ECL, electrochemiluminescence; DPASV, differential pulse anodic stripping voltammetry; LSV, linear sweep voltammetry.

**Table 3:** Electrochemical biosensors based on electrodes prepared from templated ordered porous carbon materials.

Analyte	Porous Materials		Immobilized	Electrode Configuration ^d^	Analytical Performance		Stability	Reference
	Type (Template, C Source) ^a^	Modifier ^b^	Biomolecule(s) ^c^		Concentration Range	Det. Limit		
Aflatoxin B_1_	MCNs	Thionine	AFB_1_ antibody	GCE/OMC-Thi/GluA/AFB_1_ + BSA	10–2 × 10^4^ ng∙L^−1^	3 ng∙L^−1^	20 days (100%) 50 days (92%)	[247]
Aflatoxin B_1_	MCF	Ag NPs	AFB_1_ antibody	GCE/OMC/Ag/luminol/AFB_1_ + BSA	0.1–5 × 10^4^ ng∙L^−1^	50 pg∙L^−1^	-	[248]
3-Bromobiphenyl	CMK-3 (SBA-15/sucrose)	PB-PD	Ab_2_ antibody	ITO/OMC-PB-PD/multi-HRP-DHCNTs-Ab_2_	5 × 10^−12^–2 × 10^−9^ M	2.25 × 10^−12^ M	7 days (96%) 60 days (83%)	[249]
Calmodulin (CaM)	HMPC	PAupc	Ab_1_ antibody + HRP	GCE/(OMC-chitosan)/Ab_1_ + BSA + HRP-PAupc-Ab_1_	5–10^5^ ng∙L^−1^	1.5 ng∙L^−1^	-	[250]
Cancer biomarker (EGFR)	CMK-3 (SBA-15/sucrose)	Poly(AC*-co-*MDHLA)	EGFR antigenC	AuE/(OMC + poly(AC*-co-*MDHLA)) + anti-EGFR/AMS	10–5 × 10^4^ ng∙L^−1^	3 ng∙L^−1^	-	[251]
Catechol	CMK-3 (SBA-15/sucrose)	Copper	LAC	Au/(OMC-Cu/LAC + chitosan)	6.7 × 10^−7^–1.57 × 10^−5^ M	6.7 × 10^−7^ M	30 days (95%)	[209]
Catechol	GMC (SiO_2_ nanospheres/PS)	Co_3_O_4_ nanorods	TYR	GCE/OMC-TYR-Co_3_O_4_/chitosan	5.0 × 10^−8^–1.3 × 10^−5^ M	2.5 × 10^−8^ M	2 months (86%)	[210]
Carcinoembryonic antigen	MCF	Au NPs	Ab_2_ antibody	GCE/(GO/chitosan/BSA) (OMC/Au/Ab_2_)	0.05–10^3^ ng∙L^−1^	24 pg∙L^−1^	15 days (92%)	[252]
Chlorpyrifos	OMC	Fc@MWCNTs-CS	Aptamer	GCE/(OMC-chitosan)/Fc@MWCNTs-CS/(Apt + BSA)	5–10^5^ µg∙L^−1^	0.33 µg∙L^−1^	2 weeks (96%) 4 weeks (89%)	[253]
Ethanol	CMK-3 (SBA-15/sucrose)	Meldola’s Blue	ADH	GCE/OMC-MB/(ADH + BSA + GluA)	up to 6 × 10^−3^ M	1.9 × 10^−5^ M	15 days (22%)	[211]
Ethanol NADH	GMC	Meldola’s Blue	ADH	SPE/(OMC + chitosan + MB + ADH)	5 × 10^−4^–1.5 × 10^−3^ M 1.0 × 10^−5^–4.1 × 10^−4^ M	8.0 × 10^−5^ M 1.86 × 10^−7^ M	40 days (91%)	[212]
Ethanol Glucose	CMK-3 (SBA-15/sucrose)	-	ADH GOD	GCE/OMC/(enzyme + BSA)/GluA/Nafion	3.0 × 10^−4^–1.3 × 10^−2^ M 5.0 × 10^−4^–1.5 × 10^−2^ M	1.0 × 10^−4^ M 1.5 × 10^−4^ M	1 month (91%)	[194]
Glucose	MCF (MSU-F/furfurylalcohol)	-	GOD	GCE/(OMC-GOD + Nafion)	up to 7 × 10^−3^ M	7 × 10^−5^ M	20 days	[213]
Glucose	2D-OMC (SBA-15/sucrose) 3D-OMC (FDU-5/sucrose)	-	GOD	GCE/(OMC-GOD + Nafion)	up to 7.94 × 10^−3^ M up to 9.90 × 10^−3^ M	1.0 × 10^−5^ M 1.0 × 10^−5^ M	45 days (86%)	[214]
Glucose	CMK-3 (SBA-15/furfuryl alcohol)	Iron oxide	GOD	Pt/(OMC-Fe_3_O_4_ + GOD)/Nafion	2 × 10^−4^–1.0 × 10^−2^ M	8 × 10^−5^ M	1 week (90%)	[215]
Glucose	CMK-3 (SBA-15/sucrose)	Pt NPs	GOD	GCE/(OMC-Pt + gelatin + GOD + GluA)	4 × 10^−5^–1.22 × 10^−2^ M	1 × 10^−6^ M	30 days (95%)	[216]
Glucose	CMK-3 (SBA-15/sucrose)	Pt NPs	GOD	GCE/(OMC-Pt + Nafion) imp. GOD	up to 7.94 × 10^−3^ M	1 × 10^−6^ M	27 days (31%)	[217]
Glucose	CMK-3 (SBA-15/sucrose)	Pt NPs	GOD	Au/OMC-Pt/PPy-GOD	5 × 10^−5^–3.7 × 10^−3^ M	5 × 10^−5^ M	15 days (50%)	[218]
Glucose	CMK-3 (SBA-15/sucrose)	Au NPs	GOD	GCE/OMC-Au/GOD	5.0 × 10^−5^–2.2 × 10^−2^ M	-	30 days (88%)	[219]
Glucose	FDU-15	-	GOD	GCE/(OMC-GOD + Nafion)	1 × 10^−4^–1 × 10^−3^ M	9 × 10^−5^ M	-	[220]
Glucose	FDU-15 or FDU-16 (F127-resol)	-	GOD	SPE (OMC + PHBMA)/GOD	5–100 mg∙L^−1^	-	-	[221]
Glucose	ACF	-	GOD	SPE/OMC/GOD	up to 20 mM	-	5 days (68%)	[222]
Glucose	MSCF	-	GOD	GCE/(MSCF/GOD + Nafion)	5.0 × 10^−5^–5.0 × 10^−3^ M	3.4 × 10^−5^ M	2 weeks (94%)	[223]
Glucose	IFMC	Pd NPs	GOD	GCE/(Pd@IFMC/GOD/Nafion)	5 × 10^−4^–10^−2^ M	1.9 × 10^−4^ M	2 weeks (91%)	[224]
Glucose	LMC (CaCO_3_/sucrose)	Co(salen)	GOD	GCE/OMC/Co(salen)/GOD	5 × 10^−4^–1.3 × 10^−2^ M	2 × 10^−4^ M	10 days (92%)	[117]
Glucose	MCF (MSU-F/furfuryl alcohol)	Fe_3_O_4_ NPs	GOD	CPE (OMC-Fe_3_O_4_ + GOD)	5 × 10^−4^–1.0 × 10^−2^ M	2 × 10^−4^ M	2 months (90%)	[225]
Glucose	BGMC (KIT-6/sucrose)	-	GOD	GCE/(OMC/GOD + Nafion)	up to 7.49 × 10^−3^ M	1.0 × 10^−5^ M	30 days (95%)	[226]
Glucose	CMK-3 (SBA-15/sucrose)	-	GOD	CPE (OMC + GOD)	up to 15 × 10^−3^ M	7.2 × 10^−5^ M	-	[196]
Glucose	OMC	-	GOD	µCPE (OMC + GOD + IL)	1 × 10^−5^–8 × 10^−5^ M	-	3 weeks (93%)	[227]
Glucose	MC film (SiO_2_ nanospheres/SU-8 resin)	-	GOD	Continuous OMC film on silicon wafer + GOD	5 × 10^−4^–5 × 10^−3^ M	-	-	[228] [229]
Glucose	OMC	AP-TCT	GOD	GCE/OMC film + grafted GOD	1 × 10^−3^–1 × 10^−2^ M	3.8 × 10^−5^ M	1 month (94%)	[230]
Glucose	MCF (MSU-F/furfuryl alcohol)	-	POx GOD	GCE/(OMC + Nafion + POx) GCE/(OMC + Nafion + GOD)	up to 1 × 10^−3^ M up to 8 × 10^−3^ M	- -	20 days	[231]
Glucose	HPGC (TEOS-F127- phenol-formalin)	PDA-Au NPs	GOD	HPGC membrane/PDA-Au NPs/GOD	1 × 10^−11^–1.2 × 10^−9^ M	4.8 × 10^−12^ M	1 week (91%) 1 month (76%)	[232]
Glucose Hydrogen peroxide	CMK-3 (SBA-15/sucrose)	NiFe_2_ NPs	GOD	GCE/(NiFe_2_-OMC + Nafion)/(GOD + BSA)/Nafion	4.86 × 10^−5^–1.25 × 10^−2^ M 6.2 × 10^−6^–4.27 × 10^−2^ M	2.7 × 10^−6^ M 2.4 × 10^−7^ M	2 weeks (93%) 4 weeks (95%)	[233]
Glucose Hydrogen peroxide	CNPs	FcMeOH MB	GOD HRP	GCE/CNP-FcMeOH/GOD GCE/CNP-MB/HRP	up to 6.0 × 10^−2^ M up to 1.5 × 10^−2^ M	-	-	[234]
Glucose L-lactate	CMM	Pt NPs/ PDDA	GOD LOD	GCE/Pt_30%_/PDDA-OMC/enzyme/Nafion	5 × 10^−7^–5 × 10^−5^ M	2.5 × 10^−6^ M 1.7 × 10^−6^ M	2 weeks (88%)	[235]
Glyphosate	CMK-3 (SBA-15/sucrose)	ZnS QDs	HRP	GCE/(OMC + chitosan)/ZnS QDs/HRP	1 × 10^−10^–1 × 10^−2^ M	-	-	[236]
Hg^II^	OMC	Au NPs	DNA	GCE/PANI/AuNPs/ssDNA	1 × 10^−14^–1 × 10^−6^ M	6 × 10^−16^ M	1 month (85%)	[257]
Hydrogen Peroxide	OMC (SBA-15/glucose)	PVA	Hb	GCE/(OMC + PVA)/Hb	4 × 10^−7^–8.75 × 10^−5^ M	5 × 10^−7^ M	2 weeks (95%)	[197]
Hydrogen peroxide	CMK-3 (SBA-15/sucrose)	-COOH	Hb	GCE/{Chitosan/OMC-Hb}_n_	1.2 × 10^−6^–5.7 × 10^−5^ M	6 × 10^−7^ M	30 days	[22]
Hydrogen peroxide	GMC-6 (SiO_2_ pellets/PS)	-	Hb	GCE/(OMC-Hb + Nafion)	1 × 10^−6^–1.84 × 10^−4^ M	1 × 10^−7^ M	16 days (97%)	[198]
Hydrogen peroxide	GMC-380	-	Hb	GCE/(OMC-Hb + Nafion)	1 × 10^−6^–2.67 × 10^−4^ M	1 × 10^−7^ M	21 days (90%)	[199]
Hydrogen peroxide	GMC monolith (SiO_2_ NPs/resorcinol-Fe/formaldehyde)	-	Hb	GCE/(OMC monolith fragments + DDAB + Hb)	1 × 10^−7^–6.0 × 10^−5^ M	1 × 10^−7^ M	1 week (95%)	[200]
Hydrogen peroxide	Macroporous carbon	-COOH	Cyt *c*	OMC-Cyt *c* monolith	2.0 × 10^−5^–2.4 × 10^−4^ M	1.46 × 10^−5^ M	1 month (86%)	[201]
Hydrogen peroxide	Macroporous carbon	-COOH	Hb	GCE/OMC-Hb/Nafion	1.0 × 10^−5^–8.0 × 10^−5^ M	-	weeks	[202]
Hydrogen peroxide	FDU-15	-	Hb	GCE/OMC film imp. Hb	2 × 10^−6^–3 × 10^−4^ M	8 × 10^−7^ M	30 days (90%)	[203]
Hydrogen Peroxide	OMCN (F127-resol)	-	Cyt c	ITO/(PDDA/OMC)_n_/Cyt c	5 × 10^−6^–1.5 × 10^−3^ M	1 × 10^−6^ M	20 days (82%)	[204]
Hydrogen Peroxide	Carbon aerogel (resorcinol/formaldehyde)	Ni Pd Ppy	Mb	CPE + OMC	5.0 × 10^−6^–9.75 × 10^−4^ M 3.0 × 10^−6^–8.15 × 10^−4^ M 2.5 × 10^−6^–1.06 × 10^−3^ M	1.68 × 10^−6^ M 1.02 × 10^−6^ M 0.85 × 10^−6^ M	2 weeks (>95%) 4 weeks (87%)	[205]
Hydrogen Peroxide	MCF	-	Mb	GCE/OMC-grafted Mb	3.5 × 10^−6^–2.45 × 10^−4^ M	1.2 × 10^−6^ M	20 days (95%)	[206]
Hydrogen Peroxide	FDU-15 (F127-resol)	Pt NPs	Hb	GCE/PDDA/OMC-Pt NPs/Hb/Nafion	2 × 10^−6^–6 × 10^−2^ M	1.0 × 10^−6^ M	20 days (96%)	[207]
Hydrogen peroxide	OMC	-	Hb	MCCE (OMC + Hb)	1 × 10^−6^–2.2 × 10^−4^ M	4 × 10^−7^ M	2 weeks (95%)	[208]
Hydrogen Peroxide	OMC (SBA-15/furfuryl alcohol)	Fe_3_O_4_	HRP	GCE/(OMC-Fe_3_O_4_ + HRP)	2.4 × 10^−7^–7.2 × 10^−4^ M	1.04 × 10^−7^ M	2 weeks (86%)	[237]
Hydrogen peroxide	3D-KSC	MOFs	MP-11	OMC-enzyme composite	3.9 × 10^−7^–1.7 × 10^−3^ M	1.27 × 10^−7^ M	30 days (89%)	[238]
Hydroquinone Catechol	OMC	Au NPs	TYR	GCE/OMC-Au NPs/l-lysine/TYR	4 × 10^−7^–8 × 10^−5^ M 4 × 10^−7^–8 × 10^−5^ M	5 × 10^−8^ M 2.5 × 10^−8^ M	1 month (85%)	[239]
Hydroquinone Catechol	OMC	Au NPs	TYR	GCE/(Au NPs + l-lysine)/OMC-Au NPs/TYR	1 × 10^−7^–1.1 × 10^−4^ M	-	-	[240]
Norepinephrine	OMC (TEOS-F127-resol)	-	PNMT	SPE (OMC + PHBMA)	1-500 ng∙L^−1^	0.1 ng∙L^−1^	-	[241]
Organophosphorus pesticides	OMC	Fe_3_O_4_	AChE	SPE/(OMC + Fe_3_O_4_ + chitosan)/AChE	1-600 µg∙L^−1^	0.05 µg∙L^−1^	-	[242]
Paraoxon	OMC	-	OPH	GCE/(CB + OMC)/(OPH + Nafion)	2 × 10^−7^–8 × 10^−6^ M	1.2 × 10^−7^ M	-	[243]
Paraoxon Parathion Methyl parathion	CMK-3 (SBA-15/sucrose)	-	OPH	GCE/(OMC + Nafion)/bacteria-OPH	5 × 10^−8^–2.5 × 10^−5^ M 5 × 10^−8^–2.5 × 10^−5^ M 8 × 10^−8^–3 × 10^−5^ M	9 × 10^−9^ M 1 × 10^−8^ M 1.5 × 10^−8^ M	1 month (70%)	[244]
Pb^II^	OMC	Au NPs	DNAzyme	GCE/l-lysine/OMC-Au NPs/DNAzyme	5 × 10^−10^–5 × 10^−5^ M	2 × 10^−10^ M	1 month (87%)	[258]
Penicillin binding protein 2 a	GMC	Au NPs	Ab_1_ antibody	AuE/PAMAM/(OMC-Au NPs)/Protein A/Ab_1_/BSA	25–6400 ng∙L^−1^	0.65 ng∙L^−1^	21 days (91%)	[254]
Phenol	GMC	AA-IL	TYR	GCE/(OMC + AA-IL + TYR + chitosan)	1 × 10^−7^–1.0 × 10^−5^ M	2.0 × 10^−8^ M	21 days (90%)	[245]
Prostate-specific antigen	GMC	Au NPs	PSA aptamer	PGE/(OMC-Au NPs + chitosan) /BSA/PSA aptamer	0.25–200 µg∙L^−1^	0.25 µg∙L^−1^	30 days (93%)	[255]
Prostate-specific antigen	CMK-3 (SBA-15/sucrose)	Pd-SnO_2_ & Au NPs	Ab1 & Ab2 antigens, HRP	GCE/(Pd-SnO_2_-Au NPs + Ab1) + OMC/Au NPs-MB-Ab2-HRP	0.01–100 µg∙L^−1^	3 ng∙L^−1^	15 days (96%) 30 days (89%)	[256]
Tyramine	CMK-3 (SBA-15/sucrose)	-	TYR	GE/(OMC-TYR + PDDA + TiO_2_)/Nafion	6 × 10^−6^–1.3 × 10^−4^ M	1.5 × 10^−6^ M	7 days 14 days (70%)	[246]

^a^ Abbreviations: MCNs, mesoporous carbon nanospheres; MCF, mesoporous carbon foam; CMK, carbon mesostructures at KAIST; SBA, Santa Barbara Amorphous; HMPC, honeycomb-like mesoporous carbon; GMC, graphitized mesoporous carbon; PS, polystyrene; OMC, ordered mesoporous carbon; FDU, Fudan University (in Shanghai Materials); F127, F-127 block copolymer; ACF, activated carbon fibers obtained from carbonization of electrospinned polyaniline; MSCF, mesocellular silica-carbon nanocomposite foam; IFMC, ionic liquid derived fibrillated mesoporous carbon; LMC, large mesoporous carbon; BGMC, bicontinuous gyroidal mesoporous carbon; HPGC, hierarchically porous partially graphitic carbon; CNPs, mesoporous carbon nanoparticles; CMM, carbon mesoporous material; MCWC, mesoporous carbon/whisker-like carbon; OMCN, ordered mesoporous carbon nanospheres. ^b^ Abbreviations: NPs, nanoparticles; PB, Prussian Blue; PD, polydopamine; PAupc, poly(acrylic acid)-functionalized Au popcorn; Poly(AC*-co-*MDHLA), poly-acrylamide-*co*- methacrylate of dihydrolipoic acid; Fc@MWCNTs-CS, ferrocene functionalized chitosan on multiwalled carbon nanotubes; Co(salen), cobalt(II) Schiff base; AP-TCT, aminophenyl-2,4,6-trichloro-1,3,5-triazine; PDA, polydopamine; FcMeOH, ferrocene methanol; MB, methylene Blue; PDDA, poly(diallyl-dimethyl-ammonium chloride); QDs, quantum dots; PVA, poly(vinyl alcohol); Ppy, polypyrrole; MOFs, metal-organic frameworks; AA-IL, amino acid ionic liquid. ^c^ Abbreviations: HRP, horseradish peroxidase; EGFR, epidermal growth factor receptor; LAC, laccase; TYR, tyrosinase; ADH, alcohol dehydrogenase; GOD, glucose oxidase; POx, pyranose oxidase; LOD, lactate oxidase; DNA, desoxyribonucleic acid; Hb, Hemoglobin; Cyt *c*, Cytochrome *c*; Mb, Myoglobin; MP-11, microperoxidase-11; PNMT, phenylethanolamine N-methyl transferase; AChE, acetylcholinesterase; OPH, organophosphorus hydrolase; DNAzyme, deoxyribozyme; PSA, prostate-specific antigen. ^d^ Abbreviations: Acronyms used for biomolecules are found on the left column beside this one; GCE, glassy carbon electrode; OMC, ordered mesoporous carbon; Thi, thionine; GluA, glutaraldehyde; BSA, bovin serum albumin; ITO, indium-tin oxide; PB, Prussian Blue; PD, polydopamine; multi-HRP-DHCNTs-Ab_2_, multi-horseradish peroxidase-double helix carbon nanotubes-secondary antibody; HRP-PAupc-Ab_1_, horseradish peroxidase and Ab_1_ antigen covalently bound to poly(acrylic acid)-functionalized Au popcorn; AuE, gold electrode; Poly(AC*-co-*MDHLA), poly-acrylamide-*co*-methacrylate of dihydrolipoic acid; anti-EGFR, antigen of epidermal growth factor receptor; AMS, amino-functionalized mesoporous silica; Fc@MWCNTs-CS, ferrocene functionalized chitosan on multiwalled carbon nanotubes; MB, methylene blue; PPy-GOD, electrogenerated polypyrrole in the presence of glucose oxidase; SPE, screen-printed electrode; PHBMA, poly(*tert*-butyl methacrylate); MSCF, mesocellular silica-carbon nanocomposite foam; Pd@IFMC, nanohybrid material made of Pd nanoparticles decorated ionic liquid derived fibrillated mesoporous carbon; Co(salen), cobalt(II) Schiff base; µCPE, micro carbon paste electrode (formed in the microcavity of a Pt cavity ultramicroelectrode); HPGC membrane/PDA, hierarchically porous partially graphitic carbon membrane covered with polydopamine; CNP, carbon nanoparticle; PDDA, poly(diallyl-dimethyl-ammonium chloride); QDs, quantum dots; PANI, polyanilline; ssDNA, single strand DNA; PVA, poly(vinyl alcohol); DDAB, didodecyldimethylammonium bromide; (PDDA/OMC)_n_, n bilayers of poly(diallyl-dimethyl-ammonium chloride) and ordered mesoporous carbon particles; CPE, carbon paste electrode; MCCE, mesoporous carbon ceramic electrode; CB, carbon black; PAMAM, polyamidoamine; AA-IL, amino acid ionic liquid; PGE, pyrolytic graphite electrode; GE, graphite electrode.
